# The triglyceride-synthesizing enzyme diacylglycerol acyltransferase 2 modulates the formation of the hepatitis C virus replication organelle

**DOI:** 10.1371/journal.ppat.1012509

**Published:** 2024-09-06

**Authors:** Isabelle Reichert, Ji-Young Lee, Laura Weber, Marceline M. Fuh, Lina Schlaeger, Stefanie Rößler, Volker Kinast, Sarah Schlienkamp, Janina Conradi, Florian W. R. Vondran, Stephanie Pfaender, Pietro Scaturro, Eike Steinmann, Ralf Bartenschlager, Thomas Pietschmann, Joerg Heeren, Chris Lauber, Gabrielle Vieyres

**Affiliations:** 1 Leibniz Institute of Virology (LIV), Hamburg, Germany; 2 Heidelberg University, Medical Faculty Heidelberg, Department of Infectious Diseases, Molecular Virology, Center for Integrative Infectious Diseases Research, Heidelberg, Germany; 3 German Center for Infection Research (DZIF), partner site Heidelberg, Heidelberg, Germany; 4 Department of Biochemistry and Molecular Cell Biology, Center for Experimental Medicine, University Medical Center Hamburg-Eppendorf, Hamburg, Germany; 5 Department of Medical Microbiology and Virology, Carl von Ossietzky University Oldenburg, Oldenburg, Germany; 6 Department of Molecular and Medical Virology, Ruhr University Bochum, Bochum, Germany; 7 Integrative Analysis of Pathogen-Induced Compartments, Leibniz ScienceCampus InterACt, Hamburg, Germany; 8 ReMediES, Department of General, Visceral and Transplantation Surgery, Hannover Medical School, Hannover, Germany; 9 Institute of Virology and Cell Biology, University of Luebeck, Luebeck, Germany; 10 German Cancer Research Center (DKFZ), Division Virus-Associated Carcinogenesis, Heidelberg, Germany; 11 Institute for Experimental Virology, TWINCORE Centre for Experimental and Clinical Infection Research, a joint venture between the Helmholtz Centre for Infection Research and the Hannover Medical School, Hannover, Germany; 12 Cluster of Excellence RESIST (EXC 2155), Hannover Medical School, Hannover, Germany; University of Maryland at College Park: University of Maryland, UNITED STATES OF AMERICA

## Abstract

The replication organelle of hepatitis C virus (HCV), called membranous web, is derived from the endoplasmic reticulum (ER) and mainly comprises double membrane vesicles (DMVs) that concentrate the viral replication complexes. It also tightly associates with lipid droplets (LDs), which are essential for virion morphogenesis. In particular acyl-CoA:diacylglycerol acyltransferase 1 (DGAT1), a rate-limiting enzyme in triglyceride synthesis, promotes early steps of virus assembly. The close proximity between ER membranes, DMVs and LDs therefore permits the efficient coordination of the HCV replication cycle. Here, we demonstrate that exaggerated LD accumulation due to the excessive expression of the DGAT1 isozyme, DGAT2, dramatically impairs the formation of the HCV membranous web. This effect depended on the enzymatic activity and ER association of DGAT2, whereas the mere LD accumulation was not sufficient to hamper HCV RNA replication. Our lipidomics data indicate that both HCV infection and DGAT2 overexpression induced membrane lipid biogenesis and markedly increased phospholipids with long chain polyunsaturated fatty acids, suggesting a dual use of these lipids and their possible competition for LD and DMV biogenesis. On the other hand, overexpression of DGAT2 depleted specific phospholipids, particularly oleyl fatty acyl chain-containing phosphatidylcholines, which, in contrast, are increased in HCV-infected cells and likely essential for viral infection. In conclusion, our results indicate that lipid exchanges occurring during LD biogenesis regulate the composition of intracellular membranes and thereby affect the formation of the HCV replication organelle. The potent antiviral effect observed in our DGAT2 overexpression system unveils lipid flux that may be relevant in the context of steatohepatitis, a hallmark of HCV infection, but also in physiological conditions, locally in specific subdomains of the ER membrane. Thus, LD formation mediated by DGAT1 and DGAT2 might participate in the spatial compartmentalization of HCV replication and assembly factories within the membranous web.

## Author summary

Lipid droplets are fat storage organelles with an increasingly recognized importance for the infectious cycle of multiple pathogens. In particular, they support hepatitis C virion morphogenesis and the formation of the picornaviral replication organelle. Unexpectedly, we observed that exaggerated lipid droplet biogenesis, due to an excessive expression of the triglyceride forming enzyme DGAT2, is deleterious to hepatitis C virus replication. More precisely, the formation and maintenance of the viral replication organelle was impeded. Exaggerated lipid droplet biogenesis induced by DGAT2 also profoundly altered the cell lipidome, with strong accumulation of neutral lipids and changes in the membrane lipid composition. Our results support a model where lipid flux occurring during lipid droplet biogenesis regulates the composition of the endoplasmic reticulum membranes, where lipid droplets are formed, and thereby affect the capacity of hepatitis C virus to remodel these membranes into its replication organelle. Since DGAT1 and its triglyceride synthesis were previously reported to contribute to virus assembly, this also implies that lipid droplet biogenesis driven by DGAT1 and DGAT2 but also membrane contact sites between endoplasmic reticulum and lipid droplets might locally restrict and determine the formation of hepatitis C virus replication and assembly factories.

## Introduction

Hepatitis C virus (HCV) infection is one of the leading causes of liver cirrhosis and cancer [1]. HCV isolates can display high genetic variability and are divided into 7 clinically relevant genotypes, among which genotype 1 and the highly steatogenic genotype 3 account for about 46% and 30% of all infections, respectively [2]. HCV is an enveloped plus-strand RNA virus and belongs to the family of *Flaviviridae*, which also includes flaviviruses such as the emergent mosquito-borne Zika or Dengue viruses (ZIKV, DENV) and tick-borne encephalitis virus (TBEV). Its 9.6 kb genome is flanked by 5’ and 3’ untranslated regions that are required for genome replication, translation and stability [3]. The single open reading frame encodes a long polyprotein that is cleaved by host proteases into three structural and seven non-structural proteins. The structural proteins comprise the capsid protein core and the envelope glycoproteins E1 and E2. The non-structural (NS) proteins consist of p7 and NS 2, 3, 4A, 4B, 5A, 5B, which play various roles in RNA genome replication as well as virus assembly [4].

One of the hallmarks of plus-strand RNA virus replication is the reshuffling of host intracellular membranes into a replication organelle. This compartment concentrates viral replication proteins and host co-factors while shielding the replication intermediates from detection by innate immune sensors [5,6]. Morphologically, viral replication organelles can occur either in the form of membrane invaginations/spherules as in the cases of DENV, ZIKV or TBEV [7–9] or accumulated vesicles, e.g. single, double or multi-membrane vesicles as in the HCV-induced membranous web [10–14] or in the case of coronavirus infections [15,16]. Interestingly, ectopic expression of the HCV polyprotein or even NS5A alone is sufficient to form the ER-derived double-membrane vesicles (DMVs), recognized as HCV main replication factory [10]. Also, various lipids, including phosphoinositol-4-phosphate (PI4P), sphingomyelin (SM), phosphatidic acid (PA) or cholesterol are enriched in HCV-infected cells and were identified as key components of the membranous web [17–21]. Consistently, the corresponding lipid enzymes and transporters are crucial for HCV RNA replication [17,22–25]. However, there is still a lack of understanding of the underlying changes in the ER lipid landscape that enable the different membrane permeability, fluidity and curvature properties required for DMV formation and its regulation.

Lipid droplets (LDs) are ubiquitous and dynamic organelles hijacked by HCV for viral assembly and maturation [26]. In particular, acyl-CoA:diacylglycerol acyltransferase 1 (DGAT1), a triacylglycerol synthesizing ER-resident transmembrane protein, recruits HCV core and NS5A proteins to the LD surface to initiate viral morphogenesis [27]. Consistently, the knockdown or inhibition of DGAT1 severely impairs the production of viral progeny, while targeting DGAT2, which catalyzes the same enzymatic reaction as DGAT1, does not affect HCV infection [27]. On the other hand, the host adipose triglyceride lipase (ATGL) and its co-factor α/β hydrolase domain-containing protein 5 (ABHD5/CGI-58) are important to mobilize lipids from the LDs at the HCV assembly site for the formation of the HCV lipo-viro-particle [28,29]. Lipid droplets are also tightly entangled in the membranous web [30–32] but contrarily to picornaviruses or Dengue virus [33,34], they have not yet been directly involved in HCV genome replication. Rather, their proximity to HCV replication organelle is believed to illustrate the spatial coordination between genome replication and assembly [30–32].

While testing DGAT2 as a potential LD marker, we found that increasing DGAT2 expression causes vast accumulation of LDs but strongly hampers HCV replication. Since LDs have so far been ascribed a proviral role in supporting HCV morphogenesis, we were intrigued and unraveled this phenotype. We report that excessive DGAT2 activity prevents HCV genome replication and the formation of DMVs. We attribute this effect to profound changes in the host cell lipidome and to the depletion or hijacking of specific membrane lipids. Our results indicate that LDs are not simply in physical proximity to the HCV replication organelle and supporting HCV morphogenesis but that their metabolism also determines HCV genome replication and host membrane remodeling required for the nesting of the viral replication factories.

## Results

### Increased DGAT2 expression hampers HCV replication across different genotypes

**Fig 1:**
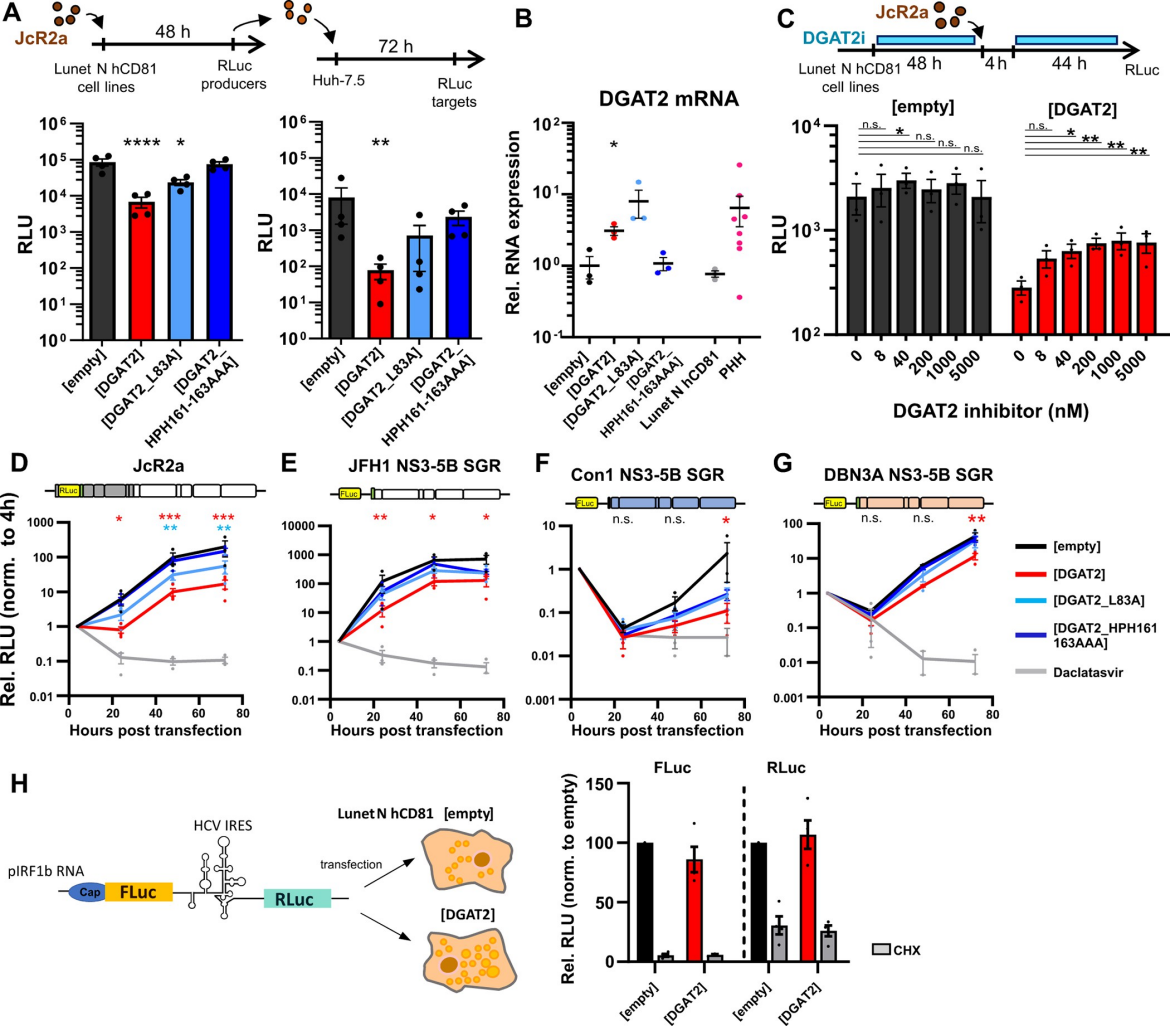
Increased DGAT2 expression hampers hepatitis C virus (HCV) infection. **(A)** Whole replication cycle assay of JcR2a. Lunet N hCD81 cells overexpressing DGAT2, DGAT2_L83A or DGAT2_HPH161-163AAA were infected with JcR2a and cell lysates were harvested 48 h post infection (hpi) for *Renilla* luciferase (RLuc) assay. The supernatant was transferred onto Huh-7.5 cells and cell lysates were harvested 72 hpi. Relative light units (RLU) of n = 2–4 were plotted. **(B)** DGAT2 mRNA expression levels in stable overexpressing Lunet N hDC81 cell lines compared to parental Lunet N hCD81 and primary human hepatocytes (PHHs) were measured by RT-qPCR. Values are normalized to the average DGAT2 mRNA expression levels in Lunet N hCD81 [empty]. Mean ± SEM of n = 3 or 8 for PHH donors are shown. **(C)** Lunet N hCD81 [empty] or [DGAT2] were treated with a DGAT2 small molecular inhibitor (DGAT2i) 48 h prior to and 4 h post JcR2a infection. Applied inhibitor concentrations ranged from 0–5 μM for DGAT2i. Viral replication was assessed by luciferase assay and values are depicted relative to the respective DMSO control (0 nM inhibitor), n = 3. **(D-G)** Full-length JcR2a **(D)**, JFH1 NS3-5B subgenomic replicon (SGR) **(E)**, Con1 NS3-5B SGR **(F)** or DBN3A NS3-5B SGR **(G)** *in vitro* transcripts (IVTs) were transfected in Lunet N hCD81 [empty] (black), [DGAT2] (red), [DGAT2_L83A] (light blue), or [DGAT2_HPH161-163AAA] (dark blue) cell lines by electroporation. The replication inhibitor Daclatasvir was added at 1 nM to transfected Lunet N hCD81 [empty] cells as a control for viral replication. Viral replication was measured by luciferase assay at 4–72 hpi. Mean values normalized to 4 hpi ± SEM are shown (n = 3). **(H)** pIRF1b dual luciferase reporter IVTs were transfected in Lunet N hCD81 [empty] or [DGAT2] cells followed by 8 h incubation in the presence or absence of 20 μM cycloheximide (CHX). Firefly luciferase (FLuc) and RLuc signals were measured and plotted normalized to Lunet N hCD81 [empty]. n = 4. Each dot in (**A, B** and **H**) represents one biological replicate. Statistical tests were performed for the individual cell lines against the [empty] **(A, B)** or untreated **(C)** control group. In **(D-G)**, significant changes of 72 h values compared to [empty] control group are shown. The asterisk color corresponds to the respective cell line.

To study the influence of DGAT2 on the replication of HCV, we utilized the hepatoma cell line Lunet N hCD81 and generated stable cell lines overexpressing human DGAT2. By using the full-length *Renilla* luciferase reporter virus JcR2a, we assessed the effect of DGAT2 expression on the whole HCV replication cycle (Fig 1A). Surprisingly, the overexpression of DGAT2 strongly reduced reporter gene expression with a 13-fold reduction in the first and a 100-fold reduction in the second round of infection as compared to the control. Mutations within a putative neutral lipid binding site (L83A) or a highly conserved acidic sequence (HPH161-163AAA), both previously shown to impair the enzymatic activity of DGAT2 [35], partially or completely alleviated this effect (Fig 1A). Note that the DGAT2- or DGAT2 mutant-expressing cell lines had slightly impaired cell growth (S1 Fig), consistent with previous reports [36]. However, this mild effect was unlikely to account for the strong HCV replication inhibition. As expected, we could confirm DGAT2 mRNA upregulation by RT-qPCR, except for the triple mutant, which could not be detected due to the impaired binding of the antisense qPCR primer at the mutated site. Remarkably, DGAT2 mRNA overexpression was mild since it did not exceed endogenous DGAT2 RNA levels within primary human hepatocytes (Fig 1B). Note that the protein abundance of endogenous or ectopically expressed untagged DGAT2 is difficult to detect due to lack of useful commercially available antibodies [37].

Next, we wondered, whether the impact of the increased DGAT2 levels on HCV replication can be reversed by DGAT2 small molecule inhibitors (DGAT2i) and, secondly, if HCV replication benefits from blocking the endogenous DGAT2 activity. To address these questions, we applied the inhibitor to our cell lines and inoculated them with JcR2a (Fig 1C). Inhibition of endogenous DGAT2 in the control cell line did not significantly affect viral replication, indicating that the endogenous levels of DGAT2 did not restrict HCV replication. However, treatment of DGAT2-overexpressing cells with the DGAT2 inhibitor partially reverted the DGAT2 antiviral phenotype and increased viral replication up to 3-fold compared to the untreated control. Together with the observed phenotype of the DGAT2 mutants (Fig 1A), these results indicate that DGAT2 enzymatic activity is important for the protein antiviral effect.

We then wanted to pinpoint the step of the HCV replication cycle hindered by the excess of DGAT2. As the overexpression of DGAT2 inhibits already the first round of HCV infection (Fig 1A), we concluded that the antiviral activity of DGAT2 most likely targets a pre-assembly step of the virus life cycle. We therefore circumvented HCV entry and monitored the replication of transfected full-length JcR2a reporter virus in DGAT2-overexpressing cells (Fig 1D). DGAT2 expression delayed the onset of JcR2a replication and decreased the replication efficiency about 100-fold at 72 h post transfection (hpt) (Fig 1D). We could reproduce this antiviral effect with the corresponding JFH1-NS3-5B subgenomic replicon (SGR) (Fig 1E). Furthermore, to investigate the HCV genotype-specificity of the DGAT2 antiviral activity, we also tested NS3-5B SGRs of genotypes 1b (Con1) and 3a (DBN3a) (Fig 1F and 1G). While the replication kinetics of all SGRs differed, we found that increased DGAT2 expression impaired the replication of all tested constructs. The replication of the highly steatogenic genotype 3a (Fig 1G) was reduced to a lesser extent (~3 to 4-fold) as compared to JFH1 SGR (Fig 1E) but statistically significant. In all cases, mutation of the lipid binding site or of the conserved HPHG motif decreased or abolished the antiviral effect of DGAT2, respectively (Fig 1D–1G). In summary, DGAT2 impairs a post-entry and pre-assembly step of HCV replication in a mechanism that is conserved across all tested genotypes.

By utilizing a dual luciferase reporter system [38], we then examined the effect of DGAT2 on the IRES-mediated translation of HCV (Fig 1H). The reporter construct allowed us to assess the IRES-mediated translation of *Renilla* luciferase and cap-controlled translation of Firefly luciferase in the cell lines of interest. The expression of DGAT2 did not impair cap- or IRES-mediated translation of the dual luciferase reporter. We therefore concluded that DGAT2 antiviral activity is probably related to a deficiency in virus genome replication.

### DGAT2 hinders the formation and maintenance of HCV replication organelle

**Fig 2:**
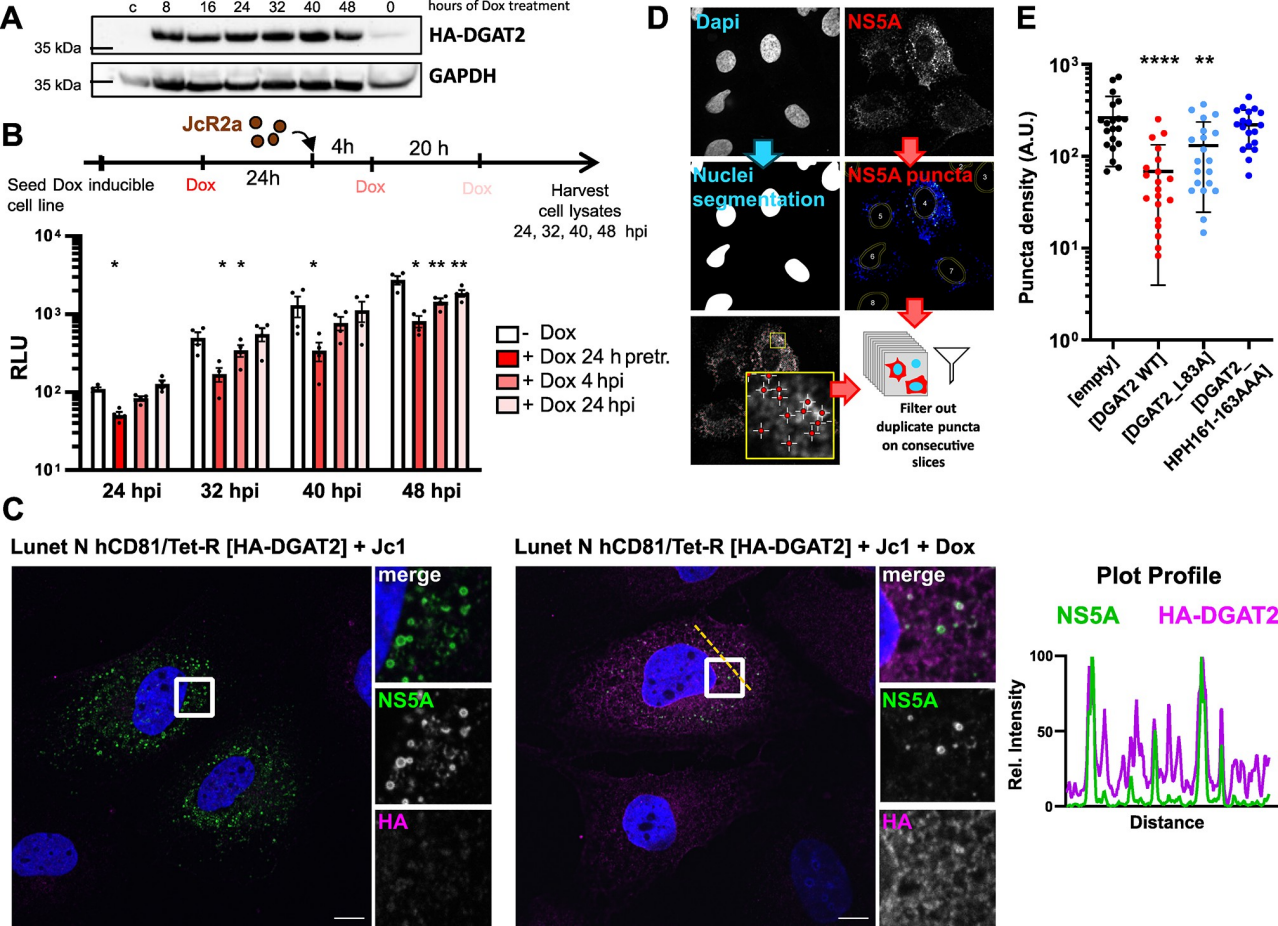
Increased DGAT2 expression impairs the formation of HCV replication factories. **(A)** Doxycycline (Dox)-inducible HA-DGAT2 Lunet N hCD81 cells were treated with Dox for the indicated time period before lysis for Western blot analysis. The first lane shows Lunet N hCD81 [empty] control cell lysate. HA-DGAT2 (~45–50 kDa) was detected by an anti-HA antibody, GAPDH (~36 kDa) was stained as loading control. **(B)** Dox-inducible HA-DGAT2 Lunet N hCD81 cells were infected with JcR2a and treated with Dox at different times prior to and post infection (untreated, 24 h prior to infection, 4 or 24 hours post infection (hpi)). Cell lysates were harvested for luciferase assay at 24, 32, 40 and 48 hpi. Each dot represents one biological replicate (n = 3). **(C)** Localization of HA-DGAT2 in HCV-infected cells. Dox-inducible HA-DGAT2 Lunet N hCD81 cells were infected with Jc1 and treated with DMSO vehicle control (left panel) or Dox (right panel) 4 hpi. The cells were fixed 48 hpi. NS5A (green) and HA-DGAT2 (magenta) were detected by immunofluorescence staining. Nuclei were stained with DAPI (blue). The white box area in the overview image (left side) is enlarged for each channel (right side). The intensity plot profile was computed along the depicted dotted yellow line. Representative images of 3 independent experiments are shown. **(D)** Automated z-stack image quantification of HCV NS5A puncta. Cell nuclei and NS5A puncta were automatically segmented. NS5A puncta were quantified in the z-stack images after excluding duplicate puncta appearing at the same position in consecutive slides of the z-stack. The number of NS5A puncta was normalized for the nuclear area of HCV-positive cells in each image field. **(E)** NS5A puncta quantification was performed in stable Lunet N hCD81 cell lines expressing DGAT2, DGAT2_L83A or DGAT2_HPH161-163AAA 48 h post transfection with JcR2a (n = 2). A.U. = arbitrary units. Results of statistical analysis are indicated by asterisks.

To further specify the timing of DGAT2 antiviral activity, we generated Lunet N hCD81 cell lines with inducible HA-tagged DGAT2 expression. We confirmed HA-DGAT2 protein expression (Fig 2A) and observed an increasing LD content upon Doxycycline treatment (S2 Fig), which was expected due to DGAT2’s role in the LD biogenesis [39]. After verifying the antiviral activity of the HA-tagged DGAT2 construct (S3 Fig), the system allowed us to study the antiviral effect of DGAT2 when induced at different times prior to and post infection (Fig 2B). Note that the used JcR2a virus has a rather low kinetics so that we do not expect reinfection to play a major role in this setting. As expected, inducing HA-DGAT2 expression prior to infection remarkably reduced the viral replication already at 24 hours post infection (hpi) as compared to the non-induced control. However, inducing DGAT2 expression 4 or 24 hpi also hampered HCV replication (starting from 32 or 48 hpi, respectively), indicating that DGAT2 expression decreases viral replication even after its onset.

To get a better understanding of the mechanism behind the DGAT2-mediated inhibition of HCV replication, we analyzed the formation of HCV replication complexes and replication organelles in DGAT2-overexpressing cells. As a marker for HCV replication complexes [40] we focused on NS5A. Using our inducible expression system, we found that NS5A puncta partially co-localized with HA-tagged DGAT2 in infected cells (Fig 2C). We also measured the formation of NS5A puncta in three-dimensional z-stacks of HCV-transfected cells (Fig 2D). In Lunet N hCD81 DGAT2-overexpressing cells, the NS5A puncta density was significantly reduced as compared to the control cells (Fig 2E). Matching our earlier results, the reduction was dependent on the catalytic activity of DGAT2, since both L83A and HPH161-163AAA mutants decreased the NS5A puncta density to a lesser extent than catalytically active DGAT2.

In this last setup however (Fig 2D and 2E), replication inhibition led to an overall decrease of the NS5A signal. This low amount of viral proteins could account for the decreased number of replication complexes, independent of the proteins capacity to form replication factories. To distinguish replication efficiency from the ability of HCV NS proteins to reshuffle membranes, we used a pTM-based expression construct encoding the HCV NS3 to NS5B polyprotein under the control of the T7 RNA polymerase [41]. This allowed us to study HCV membranous web formation in a replication-independent system. To test whether DGAT2 expression affected the formation of HCV replication organelle, we transfected Lunet T7 cells stably expressing the DGAT2 constructs with the pTM construct comprising a GFP-tagged version of NS5A, so that we could locate NS5A-positive cells by fluorescence prior to transmission electron microscopy [10].

**Fig 3:**
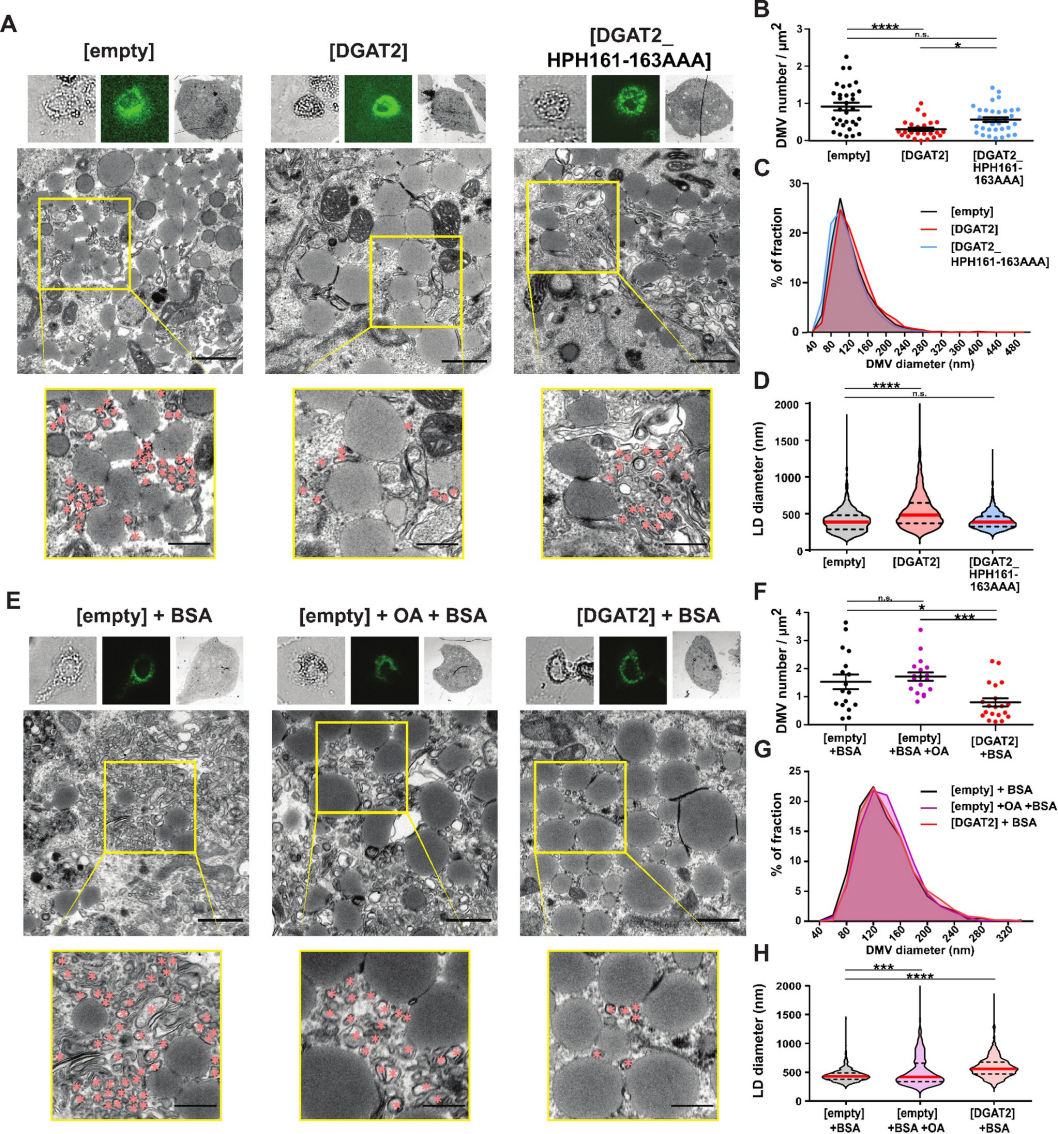
Effect of DGAT2 overexpression and oleic acid treatment on DMV formation. **(A-H)** Stable Lunet T7 cells overexpressing [DGAT2] or [DGAT2_HPH161-163AAA] were transfected with the pTM expression vector encoding HCV NS3-5B/5A^EGFP^. Cells were fixed 24 h post transfection. Transfected cells were first identified by GFP signal then fixed and further processed for CLEM analysis. In panels **(E-H)**, transfected cells were additionally treated after 18 h with BSA (30 μg/mL) or 360 μM oleic acid (OA) combined with BSA. **(A, E)** Representative images. The upper panel shows from left to right bright-field, fluorescent and electron microscopy overview images of a representative cell. The transmission electron microscopy (TEM) image in the middle panel is further enlarged in the yellow box area and depicted in the lower panel. Red asterisks indicate DMVs. Scale bar for middle image, 1 μm; for magnified image, 500 nm. **(B, C, F, G)** DMV and **(D, H)** LD profiles were analyzed using TEM images taken at x4 k magnification. **(B, F)** Number of DMVs per μm^2^, **(C, G)** size distribution of DMVs and **(D, H)** diameter of LDs in nm, respectively. Statistical tests were performed for the individual cell lines against the [empty] **(B, C, D)** or [empty]+BSA **(F, G, H)** control group. Results of statistical analysis are indicated by asterisks.

We found membrane rearrangements in the cytoplasm of transfected Lunet T7 [empty] cells, including DMVs and multi membrane vesicles (MMVs), which are typical features of the HCV membranous web (Fig 3A). In contrast, the DMV number was reduced by DGAT2, but not by DGAT2_HPH161-163AAA expression (Fig 3A and 3B). There was no major shift in the DMV size distribution visible (Fig 3C). Instead, we observed increased size of LDs in DGAT2-overexpressing cells (Fig 3A and 3D) and therefore inquired whether accumulation of large LDs physically prevented DMV accumulation. Arguing against this hypothesis, control cells fed with oleic acid (OA) which is known to stimulate LD growth [42] had unchanged DMV numbers (Fig 3E and 3F) and size distribution (Fig 3G), although they also accumulated enlarged LDs (Fig 3H). Consistently, OA treatment only mildly inhibited HCV infection at high doses (S4 Fig). We therefore concluded that the dramatic impairment of HCV membranous web formation was DGAT2-specific and not merely driven by LD accumulation. Interestingly, as described earlier [30], we observed occasional ER-enwrapped LDs in close proximity to DMVs (S5A Fig). These enwrapped LDs were even more frequent in DGAT2-overexpressing cells (S5A and S5B Fig), which points towards increased ER-LD interorganellar contact sites in the DGAT2 expressing cells.

### Excessive DGAT2 activity also inhibits Flavivirus replication

Since we related the inhibitory phenotype of DGAT2 overexpression to the formation of the HCV replication organelle, we were interested in the DGAT2-sensitivity of other RNA viruses relying on the formation of a membrane-derived replication compartment. Therefore, we tested ZIKV and Langat virus (LGTV, a close relative of TBEV), both belonging to the virus family *Flaviviridae*, as well as human common cold coronavirus (HCoV) 229E and hepatitis E virus (HEV) for their capacity to replicate in the stable DGAT2 expressing cell lines (S6 Fig). Both ZIKV and LGTV replications were significantly impaired by DGAT2 overexpression (S6A and S6B Fig). For ZIKV, the antiviral activity across the DGAT2 constructs showed a very similar trend compared to HCV replication. In contrast, DGAT2 did not significantly affect HCoV 229E or HEV infection (S6C and S6D Fig). Altogether, this indicates that DGAT2 regulates HCV replication but also more broadly the flaviviral replication cycle. Due to the stronger effects observed, we further unraveled DGAT2 antiviral effect in the HCV model.

### DGAT2 antiviral activity correlates with lipid droplet accumulation

**Fig 4:**
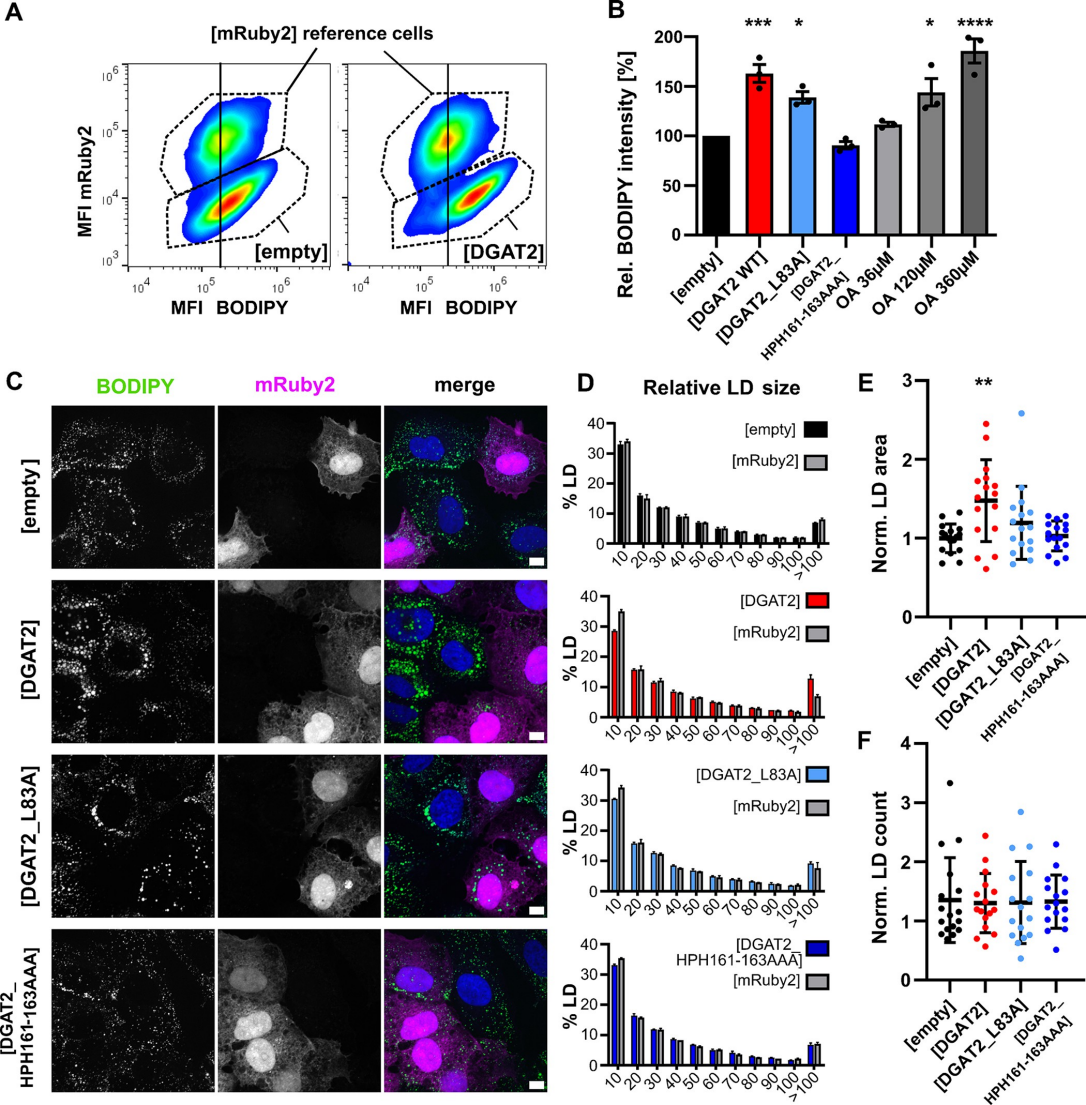
Increased DGAT2 expression increases LD size. **(A)** Example plots of flow cytometry assay to determine the LD amount in the tested cell lines. Stable Lunet N hCD81 cell lines were harvested and mixed with mRuby2-positive reference cells prior to staining with the LD dye BODIPY 493/503. BODIPY and mRuby2 signal intensities were measured by flow cytometry. To determine the LD amount in the cell lines of interest, BODIPY mean fluorescence intensity (MFI) was compared in each sample between the cells of interest (mRuby2-negative, bottom gate) and the mRuby2-positive reference cell population (top gate). The vertical line was added to highlight the shift in BODIPY signal intensity between the DGAT2-overexpressing and the reference cells, in the plot on the right. **(B)** Relative LD amount in the DGAT2-overexpressing or OA-treated (36, 120, 360 μM) cell lines. Values were normalized to [empty] control cells (n = 3). **(C)** Lunet N hDC81 cells stably expressing [empty], [DGAT2], [DGAT2_L83A] or [DGAT2_HPH161-163AAA] were co-seeded with Lunet N hCD81/mRuby2 cells. Cells were fixed and stained with BODIPY 493/503. Representative images of 2 biological repeats. **(D)** Histograms of relative LD sizes in the Lunet N hCD81 DGAT2 cell lines (colorful bars) compared to the mRuby2-positive cells (grey bars), n = 2. X-axis indicates the LD area in pixels. **(E, F)** LD area **(E)** and LD count per cell **(F)** in Lunet N hCD81 DGAT2 cell lines normalized to the mRuby2-positive cells. Each dot represents the values for one quantified image of two independent replicates (16–18 images altogether per condition). Results of statistical analysis are indicated by asterisks.

Since DGAT enzymes are rate-limiting in the TAG synthesis [39,43], we explored LD accumulation in our DGAT2-overexpressing cell lines using flow cytometry and microscopy (Fig 4). In both cases, we utilized the Lunet N hCD81/mRuby2 cells as an internal reference population as described earlier ([28,29], Material and Methods part) and stained LDs with the BODIPY 493/503 neutral lipid dye. DGAT2 expression triggered a strong LD accumulation with over 60% increase in BODIPY signal as measured by flow cytometry, an effect comparable to a high dose oleic acid (OA) induction (Fig 4A and 4B). LD accumulation upon expression of the DGAT2 mutants correlated with their antiviral effect (Fig 1A): DGAT2_L83A moderately increased the LD content while DGAT2_HPH161-163A mutant did not show any effect (Fig 4B). Interestingly, DGAT1 and 2 have been proposed to give rise to two distinct populations of LDs, with DGAT1 triggering LD formation and DGAT2 promoting LD growth. Hence, in *Drosophila* and rat hepatoma cells, many small or fewer large LDs accumulate respectively upon DGAT1 and DGAT2 overexpression [44]. We therefore wondered whether the different roles of DGAT2 and DGAT1 in HCV replication could be explained by the characteristics of the induced LDs. We quantified LD numbers and sizes in the DGAT2 cell lines by automated image analysis and co-seeded red fluorescent Lunet N hCD81/mRuby2 cells as internal reference (Fig 4C–4F). Supporting our electron microscopy observations (Fig 3D), overexpression of DGAT2 induced a remarkable shift of the LD population towards large LDs (Fig 4C and 4D), which consequently increased the total cellular LD area (Fig 4E), consistent with the flow cytometry results (Fig 4B). This effect was dependent on the catalytic activity of DGAT2, as shown by the two DGAT2 mutants (Fig 4C–4E). On the opposite, LD numbers were unchanged by DGAT2 overexpression (Fig 4F), which is consistent with the reported role of DGAT2 in LD growth rather than LD formation [44].

Taken together, overexpression of DGAT2 increased the average LD size and overall amount. This correlated with a strong decrease in HCV replication, a striking observation considering that LDs and triglyceride synthesis are usually regarded as supporting HCV infection [45,46,26,27].

### DGAT2 antiviral activity requires the protein association to the ER

**Fig 5:**
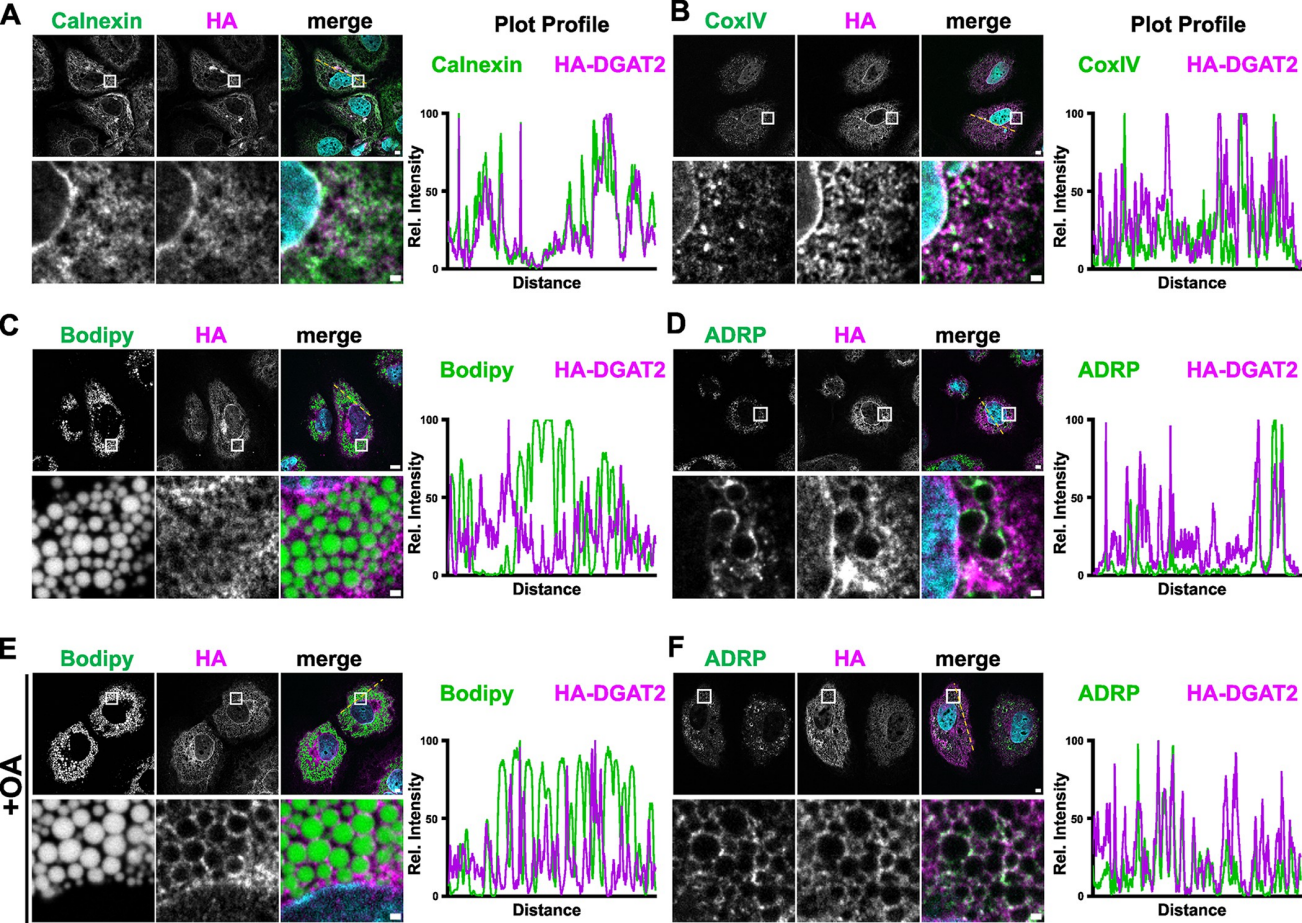
Subcellular localization of HA-tagged DGAT2. Lunet N hCD81 cells were transduced with lentiviruses to express HA-tagged DGAT2 **(A-F)**. In **(E)** and **(F)**, cells were treated with 360 μM oleic acid (OA) 6 h prior to fixation. HADGAT2 was detected with anti-HA immunofluorescence staining and depicted in magenta. ER (Calnexin, **(A)**), mitochondria (CoxIV **(B)**) and LDs (BODIPY 493/503 **(C, E)**, ADRP **(D, F)**) were co-stained and depicted in green. Nuclei were stained with DAPI and depicted in blue. The white box area in overview images (upper row of images in each panel) is enlarged in the lower row. Intensity plot profiles were computed along the depicted dotted yellow line. Representative images of at least 3 independent experiments are shown.

While endogenous DGAT2 is difficult to detect due to its low cellular abundance and lack of useful antibodies [37], overexpressed DGAT2 has been reported to localize at the ER and in close proximity to LDs and mitochondria [35,47]. We verified the localization of HA-tagged DGAT2 at these organelles in Lunet N hCD81 cells by counterstaining ER (Calnexin), mitochondria (CoxIV) and LDs (neutral lipids, ADRP) (Fig 5). While association of HA-tagged DGAT2 with the ER and enrichment at the nuclear envelope were clearly observed (Fig 5A), localization at mitochondria was more difficult to detect in our hands (Fig 5B). The localization of HA-tagged DGAT2 at LDs, leading to the characteristic ring structures, was most pronounced in cells which had been fed with OA, supporting previous findings (Fig 5C–5F) [47].

**Fig 6:**
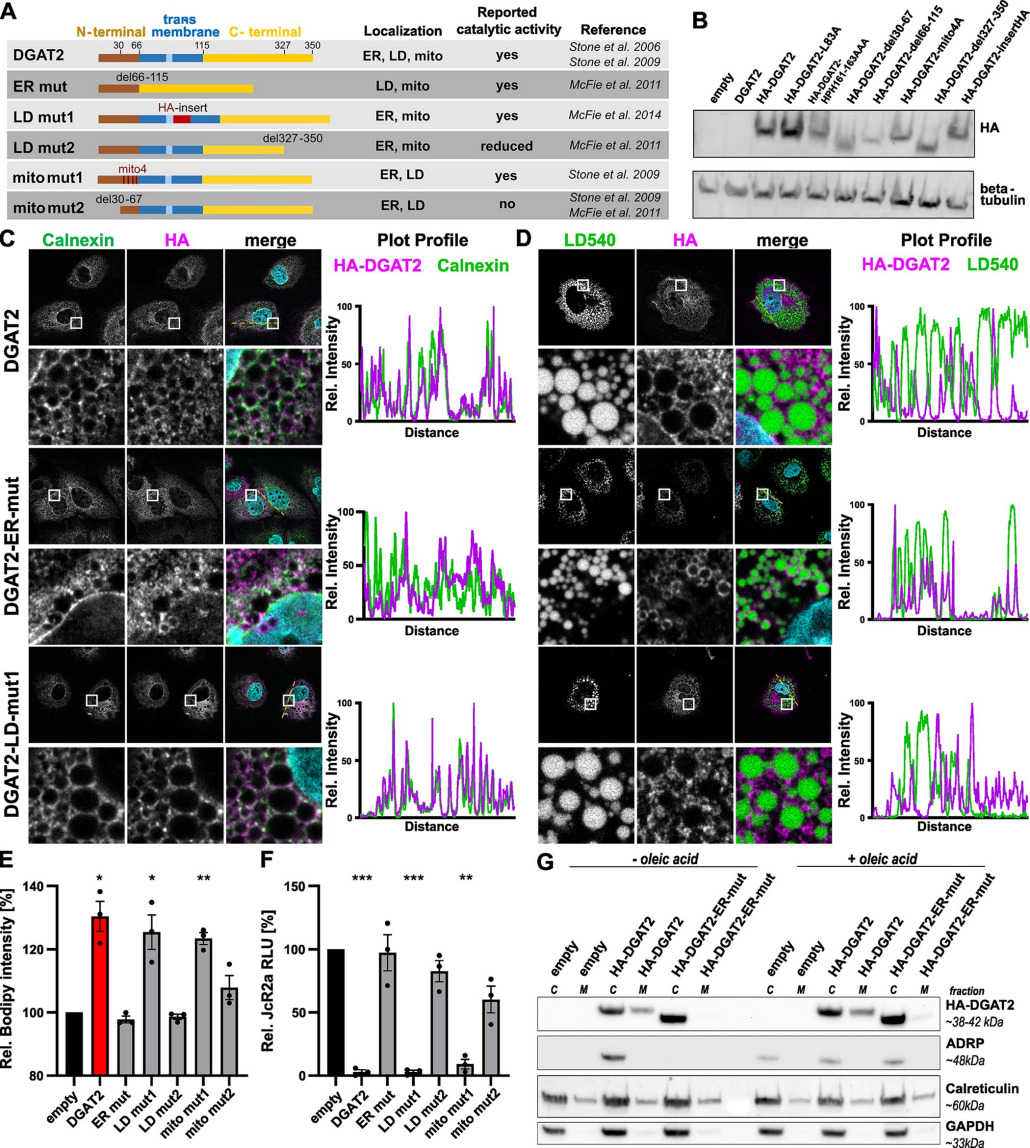
Effect of subcellular localization on the antiviral activity of DGAT2. **(A)** A panel of previously described HA-tagged DGAT2 mutants comprising variants deficient in ER (ER mut), LD (LD mut1 and LD mut2) or mitochondria (mito mut1, mito mut2) association was cloned and transduced in Lunet N hCD81 FLuc cells. 48 h post transduction, we analyzed the protein expression **(B)**, localization **(C, D)**, and effect on LD accumulation **(E)**, or infected the cells with JcR2a to measure the effect on HCV at 72 hours post infection **(F)**. **(B)** Protein expression of HA-tagged DGAT2 mutants tested by Western blot analysis. HA-tagged DGAT2 constructs (~35–50 kDa) were detected by an anti-HA antibody, beta-tubulin (~55 kDa) was stained as loading control. The first and second lanes correspond to the control and untagged [DGAT2]-overexpressing cells, respectively. **(C, D)** Localization of HA-tagged DGAT2 detected by immunofluorescence. Cells were treated with 100 μM OA overnight prior to fixation. HA-DGAT2 mutants were identified with an anti-HA antibody (magenta). ER (Calnexin **(C)**) or LDs (LD540 **(D)**) were co-stained (green). Nuclei were stained with DAPI (blue). The white box area in overview images (upper panel) is enlarged in the second row for each channel. Intensity plot profiles were computed along the depicted dotted yellow line. Representative images of at least 3 independent experiments are shown. Note that the immunofluorescence images of the remaining DGAT2 localization mutants are depicted in S7 Fig. **(E)** Relative LD content in Lunet N hCD81 cells expressing the DGAT2 mutant panel. LDs were stained with BODIPY 493/503 and LD content was measured by flow cytometry utilizing spiked in [mRuby2] reference cells (see Figs 4A and 5B). Values were normalized to [empty] control cells (n = 3). **(F)** Effect of DGAT2 mutants on HCV replication. Lunet N hCD81 cells expressing HA-tagged DGAT2 constructs were infected with JcR2a 48 h post transduction. Cell lysates were harvested 72 h post infection and viral replication was measured by luciferase assay (n = 3). Results of statistical analysis are indicated by asterisks. **(G)** Identification of HA-DGAT2 and DGAT2 ER mut in cytoplasmic (annotated C above the blots) and microsomal fractions (annotated M) with and without OA treatment. HA-DGAT2 variants were detected by anti-HA antibody (~35–42 kDa). ADRP, Calreticulin and GAPDH were detected to control for the purity of the microsomal fractions.

We then wondered whether the subcellular localization of DGAT2 plays a role in the antiviral activity. Although the tertiary structure of DGAT2 has not been solved until now, several studies identified protein domains responsible for the mouse DGAT2 ortholog association with different organelles [35,47,48]. We generated the corresponding HA-tagged human DGAT2 mutants (Fig 6A), validated their expression by Western blot (Fig 6B) and inspected their association with the ER and LDs (Fig 6C–6D). Note that we did not analyze their mitochondrial localization further, since it was not clear in our hands (Fig 5B), but still tested mutations reported to abrogate this association [47]. We also assessed the ability of DGAT2 mutants to drive LD accumulation (Fig 6E) and to inhibit HCV replication (Fig 6F). DGAT2 del66-115 mutant (ER mut) did not localize to the ER or nuclear envelope, as observed by microscopy (Fig 6C) and validated by subcellular fractionation (Fig 6G) and was inactive in terms of LD accumulation (Fig 6E) or HCV inhibition (Fig 6F), although the mouse homolog of this mutant was catalytically active *in vitro* [48]. Moreover, we identified two DGAT2 mutants (LD mut1 and mito mut1) that efficiently induced LD accumulation (Fig 6E), showed similar ER accumulation as compared to the wildtype (WT) (Fig 6C), but lacked association with LDs (Fig 6D) or mitochondria (as suggested in [47]). Expression of these mutants potently inhibited HCV replication (Fig 6F), which supports the hypothesis that the DGAT2-mediated LD accumulation is a prerequisite for the inhibition of HCV replication while LD or mitochondria association is not. The other DGAT2 mutants (LD mut2, mito mut2) did not or only partially (mito mut2) impair HCV replication (Figs 6F and S7). Based on these findings, we concluded that both the ability to trigger LD accumulation and the localization at the ER but not at the LD are crucial for the inhibitory effect of DGAT2 on HCV replication.

### Increased DGAT2 expression relocalizes DAG stores

**Fig 7:**
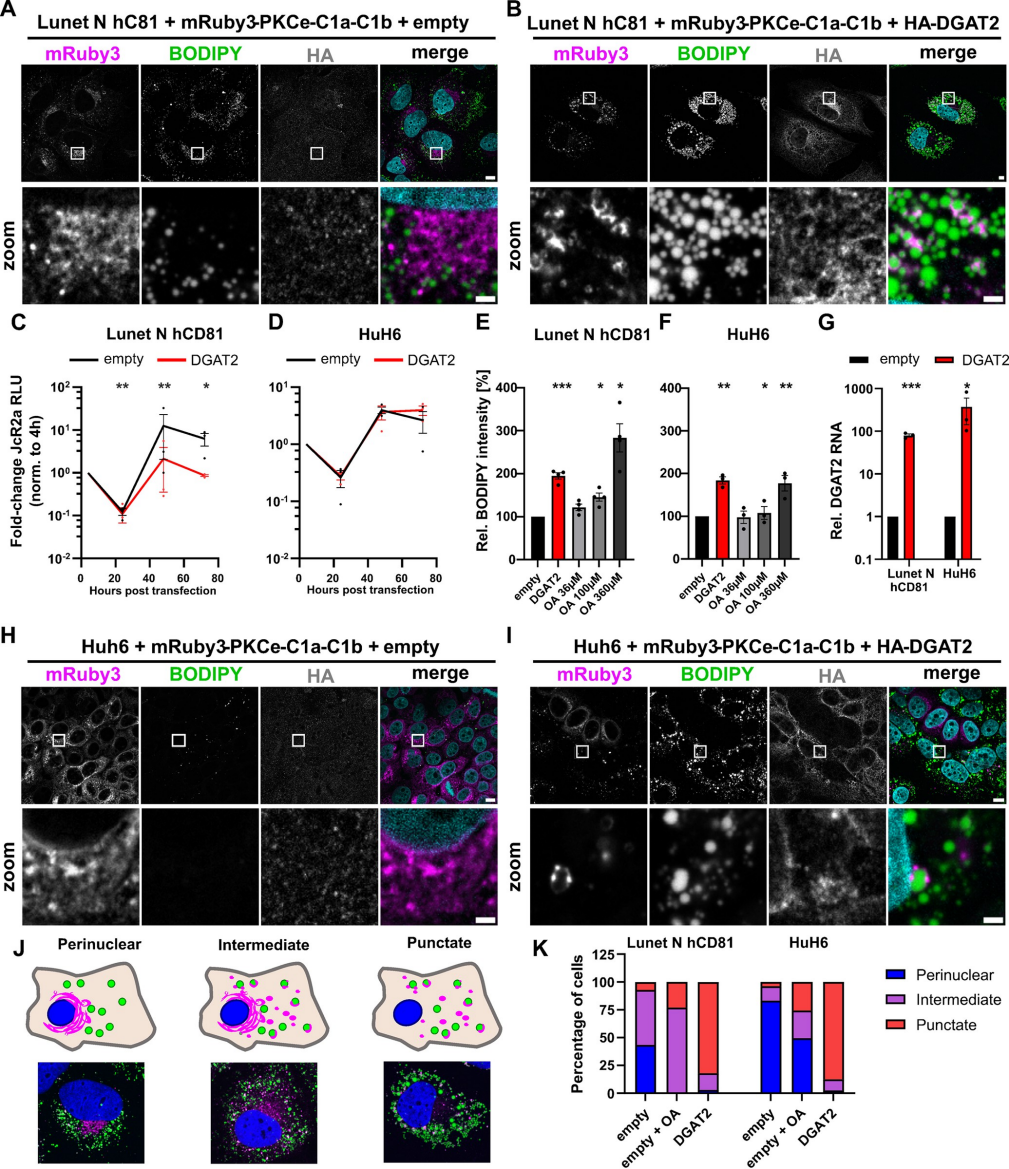
Increased DGAT2 overexpression relocalizes DAG stores. A DAG sensor, consisting of the DAG-binding domain C1a-C1b of protein kinase C epsilon (PKCe-C1a-C1b) fused to the mRuby3 fluorophore [50], was co-transduced with [empty] **(A)** or [HA-DGAT2] **(B)** in Lunet N hCD81 cells. The DAG sensor is depicted in magenta. HA-tagged DGAT2 was detected with an anti-HA antibody. LDs were stained with BODIPY 493/503 (green) and nuclei with DAPI (blue). The white box area in overview images (upper panel) is enlarged in the second row for each channel. Representative images of 3 independent experiments are shown. **(C-G)** Lunet N hCD81 **(C, E and G)** or HuH6 cells **(D, F and G)** were transduced with lentiviruses to express [empty] or [DGAT2]. **(C, D)** 48 h post transduction, cells were transfected with JcR2a *in vitro* transcripts using lipofectamine. Viral replication was measured by luciferase assay 4, 24, 48 and 72 hours post transfection (hpt). Mean ±SEM values normalized to 4 hpt are depicted (n = 3). Significant changes after log-transformation are indicated by asterisks. **(E, F)** LD content 48 h after lentiviral transduction measured by flow cytometry. OA (36, 100 or 360 μM) was added to mock-transduced cells as positive control (n = 3). **(G)** Relative DGAT2 mRNA expression 48 h after lentiviral transduction. Values were normalized to [empty] control cells (n = 2–3). **(H, I)** DAG sensor localization in HuH6 cells co-transduced with [empty] **(H)** or [HA-DGAT2] **(I)**. Fluorescence staining and imaging was performed as in **(A, B)**. Note that in panel **(I)** one can readily distinguish DGAT2-transduced from non-transduced cells based on the HA staining and that the successfully transduced cells show strong LD accumulation and a different DAG pattern. **(J, K)** Different DAG sensor localization patterns were observed in Lunet N hCD81 or HuH6 cells upon expression of [empty] or [HA-DGAT2] or OA induction. We classified the observed patterns in perinuclear or punctate localization, as well as an intermediate phenotype. **(K)** The occurrence of the individual phenotypes was manually counted in images of three independent experiments.

By esterifying DAG to TAG and connecting to numerous other lipid metabolism pathways [49], DGAT2 plays an important role in the host cell lipid metabolism. Since we could relate DGAT2 antiviral activity to the HCV DMV formation, we hypothesized that DGAT2 altered the lipid content of the host ER membrane in a way that it cannot easily be reshuffled during HCV infection. DAG is the substrate of DGAT2 and an important precursor for both triacylglycerides (TAGs) and phospholipids (PLs). Therefore, and although DAG has to our knowledge not been involved in HCV replication, we next investigated the effect of DGAT2 on DAG stores using a specific biosensor [50]. After verifying its functionality by comparison with a mutant version (S8 Fig) we tested the localization of the sensor in the HA-tagged DGAT2-overexpressing cells (Fig 7A and 7B). Strikingly, upon DGAT2 expression in Lunet N hCD81 cells, the DAG-sensor relocalized from the perinuclear region (Fig 7A and 7J–7K) to form speckles in the cytoplasm (Fig 7B and 7J–7K). Interestingly, some of these speckles localized around LDs (Fig 7B). Across this study, we identified HuH6 cells as a hepatoma cell line, in which DGAT2 overexpression does not hamper HCV replication, despite LD induction (Fig 7C–7G), suggesting slight differences in lipid metabolic pathways or rates between these cell systems. We therefore hypothesized that the localization of the DAG sensor upon DGAT2 overexpression would differ in HuH6 cells as compared to Lunet N hCD81 cells, if DAG relocalization was critical for the antiviral effect of DGAT2. However, we could clearly see the same effect of DGAT2 overexpression on the localization of the DAG sensor in the HuH6 as compared to the Lunet N hCD81 cells (Fig 7H–7K), suggesting that DAG relocalization was not the key determinant of DGAT2 antiviral phenotype.

### Excessive DGAT2 activity reshuffles the host cell lipid landscape

**Fig 8:**
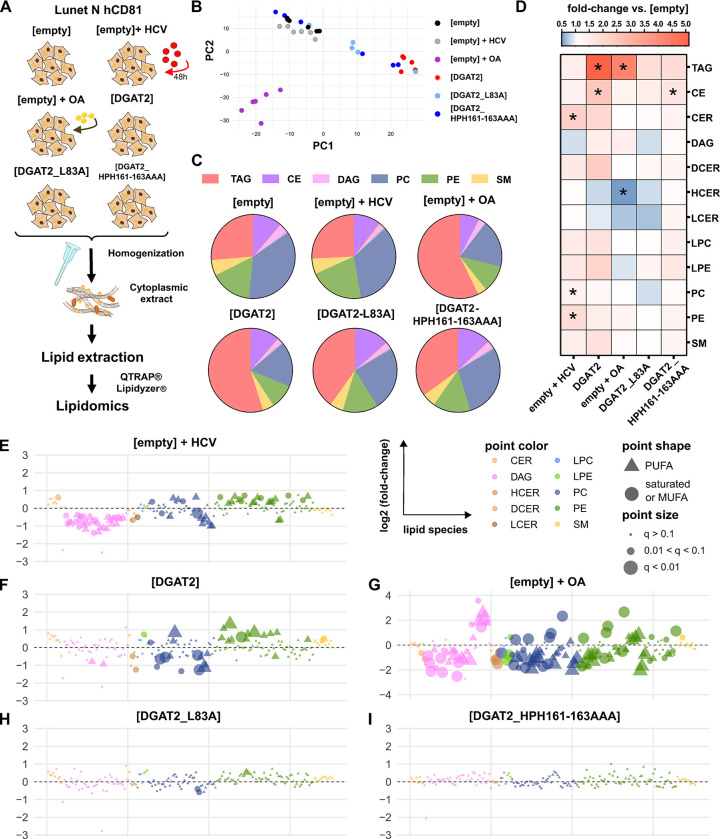
Global lipid profile changes upon HCV infection and DGAT2 expression. **(A)** Lunet N hCD81 [empty] cells, either untreated, infected with HCV Jc1 for 48 h, or treated with oleic acid, as well as Lunet N hCD81 [DGAT2], [DGAT2_L83A] or [DGAT2_HPH161-163AAA] were harvested and lysed by Dounce homogenization. Lipids were extracted and concentrated from cytoplasmic extracts and lipidomics analysis was performed using the Lipidyzer platform. Analysis of n = 6 for each condition. **(B)** Relative proportions (pie-charts) of lipid classes in the whole lipidome of cytoplasmic extracts of the different tested cell lines. **(C)** Unsupervised principal component analysis (PCA) of the lipid spec of the different conditions. Lipid concentrations were normalized to the total lipid content prior PCA. Missing values were set to 0 and data was center-scaled prior PCA. **(D)** Heat map of fold changes at the lipid class level in infected, DGAT2-overexpressing or 360 μM OA-treated cells relative to [empty]. Significant changes are indicated by asterisks (p<0.05). TAG, triacylglycerol; CE, cholesterol ester, CER, ceramide; DAG, diacylglycerol; DCER, dihydroceramide; HCER, hydroxyceramide; LCER, lactosylceramide; LPC, lyso-phosphatidylcholine; LPE, lyso-phosphatidylethanolamine; PC, phosphatidylcholine; PE, phosphatidylethanolamine; SM, sphingomyelin. **(E-I)** Bubble plots of log2 fold changes at the lipid species level in Jc1-infected, OA-treated or DGAT2-overexpressing cells relative to control [empty] cells. Values were normalized to the total membrane lipid concentration of each sample. The point color represents individual lipid classes. The point size represents the corrected p-value (q-value).

To gain a broader overview of possible lipid changes responsible for DGAT2 antiviral phenotype, we next used the Lipidyzer platform to analyze lipid concentrations of 13 lipid classes including phospholipids, sphingolipids, glycerolipids, sterols and free fatty acids. We examined the lipidome of the cytoplasmic extracts of Lunet N hCD81 cells expressing DGAT2 or infected with HCV for 48 h (Fig 8A and 8B) and analyzed differences at the levels of the lipid classes (Fig 8C–8D) and species (Fig 8E–8I). Thereby, we detected 859 lipid species in 6 biological replicates per condition. Note that we excluded free fatty acid species for technical reasons and considered 667 lipid species for further analysis, which were measured in more than 75% of the samples. Unsupervised principal component analysis (PCA) overall confirmed the clustering of our replicates and the intermediate phenotypes obtained for the DGAT2 mutants. It also clearly segregated the OA treatment control from the other conditions (Fig 8B). At the lipid class level (Fig 8C and 8D), HCV infection led to a substantial increase in membrane lipids except for DAG. Accumulations of ceramide (CER), phosphatidylcholine (PC) and phosphatidylethanolamine (PE) classes were statistically significant. These observations nicely corroborate the lipidomic changes reported by Hofmann *et al*. [20] and Diamond *et al*. [51] in Huh-7.5 cells. As expected, DGAT2 expression and OA induction led to a drastic increase of the TAG neutral lipid class, the main lipid stored in LDs. Furthermore, similar to HCV infection, various membrane lipid classes were upregulated through DGAT2 overexpression, although not statistically significant at the lipid class level. These effects were attenuated for the DGAT2 mutants, indicating that they relied on DGAT2 catalytic activity. As an exception, DAGs were regulated in an opposite direction by HCV infection and DGAT2 overexpression.

At the lipid species level (Fig 8E–8I) and focusing on membrane lipids, we observed drastic increases of CER, PC and PE, but decreased DAG lipid species in the HCV-infected cells (Fig 8E). The membrane lipid species profile of DGAT2-overexpressing cells seemed overall less affected (Fig 8F). However, we found significant trends for specific PC and PE species and observed that especially lipids with polyunsaturated fatty acyl (PUFA) chains were upregulated, whereas the amounts of saturated and monounsaturated lipids were mostly reduced upon DGAT2 overexpression. These trends were attenuated in DGAT2_L83A expressing cells and blurred for the DGAT2_HPH161-163AAA mutant (Fig 8H and 8I). Importantly, OA induction had a completely different effect on the membrane lipid profile and mostly enhanced monounsaturated fatty acyl-phospholipids (MUFA-PL) whereas several PUFA-PLs were downregulated (Fig 8G).

**Fig 9:**
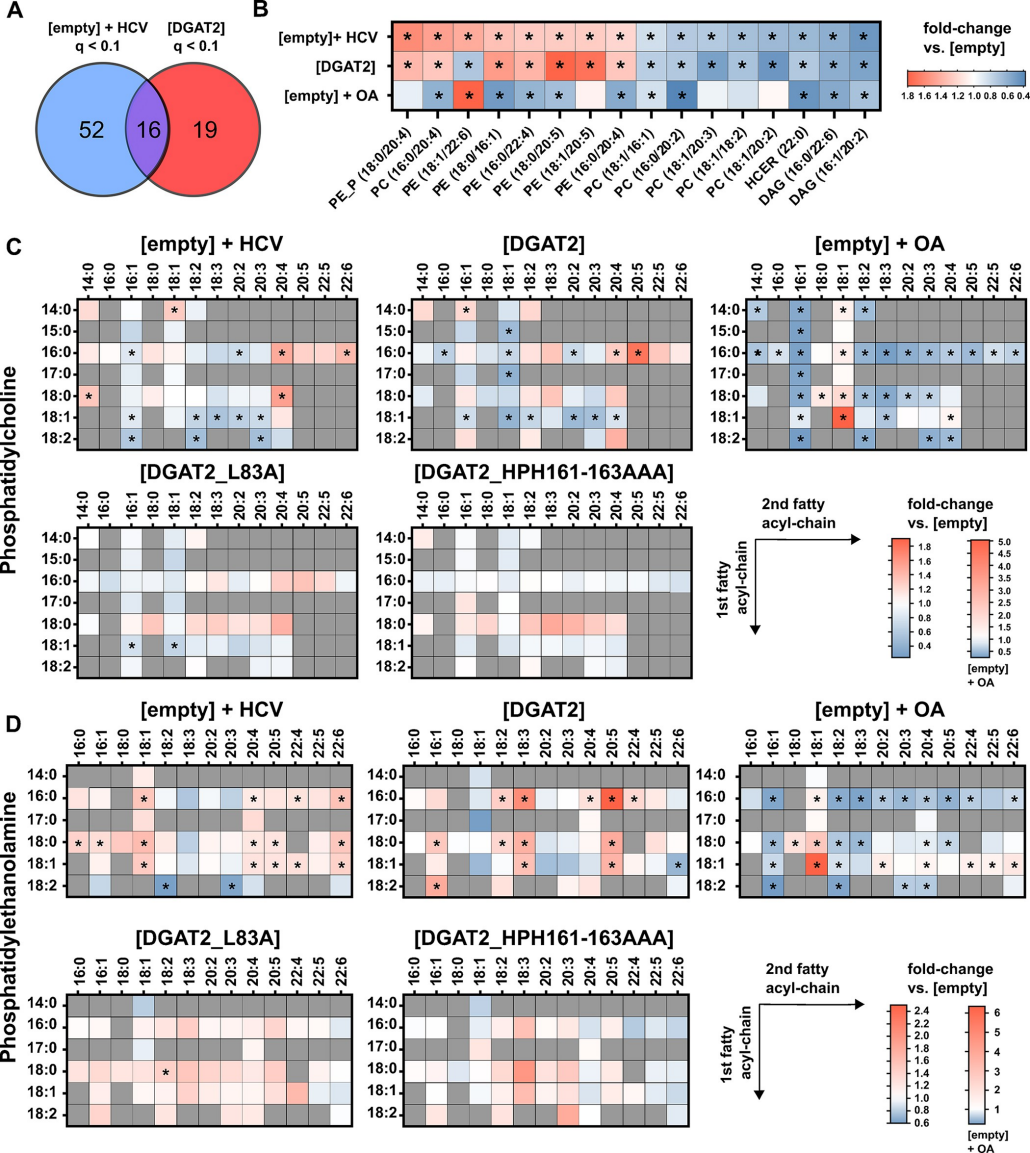
Changes of PC and PE lipid species on fatty acid subspecies level upon HCV infection and DGAT2 expression. **(A)** Within 185 measured membrane lipid species, we identified 87 lipid species that were significantly (q-value < 0.1) up- or down-regulated upon HCV infection (52 lipid species, blue) or DGAT2 expression (19 lipid species, red) or both (16 lipid species, purple) in Lunet N hDC81 cells compared to the control cells. **(B)** Fold changes of significantly regulated membrane lipid species in Jc1-infected, DGAT2-overexpressing or OA-treated cells relative to control [empty] cells are depicted in a heatmap. Note that the heatmap shows the 16 common significantly regulated lipid species of HCV-infected and DGAT2-expressing cells. Significant changes (q-value < 0.1) are highlighted by asterisks. **(C)** PC and **(D)** PE lipid species profiles in Lunet N hCD81 cells upon HCV infection, DGAT2 overexpression or OA treatment. The samples and data are the same as in Fig 8, here analyzed for single lipids on fatty acid subspecies level. Fold changes in Jc1-infected or DGAT2-overexpressing cells relative to control [empty] cells are depicted in heatmaps. Significant changes (q-value < 0.1) are highlighted by asterisks.

Overall, HCV infection and DGAT2 overexpression significantly regulated 68 and 35 lipid species, respectively (Fig 9A). Among the 16 lipid species significantly regulated in both conditions, the majority (15) was significantly regulated in the same direction (Fig 9B). Given the abundance of PC and PE in cell membranes (58 and 26% of the membrane lipids in our Lunet N hCD81 cell line, Fig 8C) and their importance in membrane biogenesis, as well as the observed trends of saturated and MUFA- versus PUFA-lipid species (Fig 8E–8I), we then analyzed the differential regulation of these lipid classes on the fatty acid subspecies level, within the membrane lipids (Fig 9C and 9D). In line with the observations made by Hofmann *et al*. [20], we found that HCV infection strongly increased PLs with long chain PUFAs, including (C20:4), (C20:5), (C22:5) and (C22:6) fatty acyl lipids in both PC and PE species. Interestingly, DGAT2 overexpression also increased the above mentioned PUFA-PLs, especially eicosapentaenoyl (EPA, C20:5) fatty acyl harboring lipids. This effect was attenuated for the L83A mutant and nearly disappeared for the triple mutant. Additionally, and in contrast to HCV infection, DGAT2 overexpression upregulated (C18:3) fatty acyl -containing PEs, but downregulated (C18:1) fatty acyl-containing PCs. Most strikingly, the highly abundant PC [18:1/18:1] species was 2.39 times reduced in DGAT2-overexpressing cells compared to the control (Fig 9C). Of note, as expected, PC [18:1/18:1] was strongly upregulated in OA-treated cells, where levels of (C18:1) in general greatly increased due to the higher abundance of the oleyl fatty acid. The fatty acyl incorporation into PLs is dependent on the overall abundance of specific fatty acid species and on the substrate preferences of the involved lipid metabolizing enzymes and transporting proteins [52]. The changes observed suggest that fatty acyl substrate channeling differs between HCV-infected and DGAT2-overexpressing cells, as a result of the substrate specificity of DGAT2 [53,54] or indirectly via the regulation of other metabolic pathways.

In conclusion, we found a large overlap of the lipid profile changes between HCV infection and DGAT2 overexpression, apart from specific lipid and fatty acyl species, suggesting that depletion of some lipid species and competition for others may explain how excessive DGAT2 activity inhibits the formation of the HCV replication organelle. Altogether, our results evidence that lipid droplet biogenesis can affect the lipid composition of intracellular membranes and thereby their reshuffling into the HCV replication organelle. As detailed in the discussion, these findings open new perspectives to understand the effect of pathological LD accumulation on virus infections but also to comprehend the spatial coordination of LD biogenesis and replication organelle formation at the suborganellar level.

## Discussion

Lipid droplets play an essential role in the production of the HCV progeny, and infection with HCV leads to an increase in LD content *in vitro* but also *in vivo* with the development of steatohepatitis [55]. Here we showed for the first time that vast accumulation of LDs in hepatocytes mediated by the overexpression of DGAT2 is deleterious for HCV replication and we linked this inhibition to a defect in membranous web formation.

Entangled within the convoluted membranes of the membranous web [30], LDs and their stored neutral fat content are hijacked by HCV to participate, together with ER and DMVs, in the assembly platform of the lipo-viro-particle [26,45]. In particular, we previously reported that the ATGL lipase and its co-factor ABHD5/CGI-58 mobilize the TAGs stored within LDs to promote particle production [28,29]. However, also the lipogenesis enzyme DGAT1 plays an essential role in HCV assembly. In fact, its expression is crucial for the recruitment of both HCV core and NS5A proteins to the LD surface [27,56], an event that is essential to initiate viral assembly [45,46]. In this context, we were surprised to find that overexpression of its isozyme DGAT2 inhibited HCV infection (Fig 1A). Mechanistically, we could relate the antiviral phenotype of DGAT2 overexpression to inefficient HCV genome replication (Fig 1D–1H), and more precisely, to a defect in membranous web formation (Fig 3). Importantly, the maintenance of HCV replication organelle was also hindered by DGAT2 overexpression, since ongoing replication could be inhibited after subsequent induction of DGAT2 (Fig 2B). Altogether, these findings underline the importance of functional LD biosynthesis for the formation and maintenance of the HCV replication organelle.

The difference between the role of DGAT1 in HCV assembly [27,56] and the detrimental effect of excessive DGAT2 activity on HCV replication is intriguing. DGAT1 and 2 are evolutionarily unrelated and although both catalyze the synthesis of TAGs from DAGs, they differ in subcellular localization and in substrate specificity since they preferentially use different DAG species in term of isomerism or fatty acid content [53,54]. It was also proposed, based on studies in the *Drosophila* model, that DGAT1 and DGAT2 give rise to separate LD subsets [57,58]. In the currently prevailing model, ER-resident DGAT1 mediates LD formation while recruitment of DGAT2 on a subpopulation of these initial LDs promotes their growth and maturation into expanding LDs. Consistently, in *Drosophila* and mammalian cells, overexpression of DGAT1 and 2 leads to an accumulation of small and large LDs, respectively [44]. In fact, we did observe an increase in large LDs upon DGAT2 overexpression, while the LD number was not affected (Fig 4C and 4D). Also, the involvement of DGAT1 in the initial steps of LD formation nicely fits with the model proposed by Herker *et al*. where DGAT1 supports the translocation of HCV core and NS5A proteins from the ER to the LD [27,56]. The role of DGAT1 in HCV assembly, the detrimental effect of excessive DGAT2 activity on HCV replication and the close proximity between replication and assembly factories in HCV-infected cells [30] suggest that HCV might have evolved to use DGAT1- rather than DGAT2-generated LDs as a platform for assembly. Further studies would be required to test this hypothesis.

DGAT2 antiviral effect correlated over a range of mutants (Figs 1, 4 and 6) and kinetically (Figs 2 and S2) with LD accumulation. Furthermore, impeding DGAT2 catalytic activity by mutations or with a small molecule inhibitor prevented or attenuated the protein antiviral effect (Fig 1), suggesting that the catalytic activity of DGAT2 rather than its potential protein-protein interactions determined the observed phenotype. Therefore, our first idea was that through excessive DGAT2 expression, the balance of neutral vs. phospholipids might be altered, which could interfere with the high demand of membrane building blocks required for the HCV membranous web formation. In other words, since the DGAT2 substrate, DAG, is also a substrate for phospholipid synthesis, excessive DGAT2 activity might divert the flux of DAG and fatty acids from phospholipid toward TAG synthesis. The differential sensitivity of other plus-strand RNA viruses to DGAT2 excessive activity (S6 Fig), despite their dependence on membrane biogenesis, was a first argument against this hypothesis. Yet, looking at the total lipid class distribution, one can easily recognize a completely altered share of neutral (TAG and cholesterol ester) vs. membrane lipids in the DGAT2-overexpressing cells (Fig 8C). However, we found a very similar partitioning of lipid classes with a tremendous increase of TAG contents upon OA feeding, which itself did not lead to DMV formation impairment (Fig 3) nor to HCV replication inhibition (S4 Fig). The fact that OA treatment triggered LD accumulation as efficiently as DGAT2 overexpression (Fig 4B) but did not strongly inhibit HCV RNA replication (S4 Fig) also indicates that mere spatial constraints, a possible boost in innate immune responses [59] or the newly reported mechanical stress sensing function of LDs [60] are not sufficient to explain DGAT2 antiviral phenotype. It is therefore likely that changes within the ER membrane lipid composition rather than simply the accumulation of neutral lipids within LDs are responsible for the DGAT2-mediated membranous web alteration. Given that both LDs and HCV replication organelle originate from the same compartment, the ER, we thought that the lipid flux from the ER membrane to the LDs during their biogenesis or expansion might affect the lipid landscape of the ER and thereby the reshuffling of this organelle necessary for productive HCV infection. Consistently, DGAT2 localized to several organelles within the cells (Fig 5), but only ER association was crucial for its antiviral phenotype (Fig 6). ZIKV and LGTV, two flaviviruses using the ER to build their replication organelle, were also sensitive, although to a lesser extent, to DGAT2 expression (S6 Fig). Why the tested coronavirus, also replicating on ER-derived membranes, was not affected (S6C Fig) is unclear to us but might have to do with virus-specific lipid requirements and fine differences in the architecture of the replication organelles. Thus, by deciphering the mechanism behind the antiviral effect of DGAT2 we hoped to get a better understanding of the lipid requirements to build the HCV replication organelle.

The formation of DMVs and MMVs as HCV replication organelle is based on a highly complex membrane folding process that requires extensive remodeling of the ER lipid landscape. In addition to forming replication organelles, ER membranes in infected cells are selectively recruited around lipid droplets and this enwrapping is enhanced at sites where both assembly and replication complexes are present [30]. Intriguingly, DGAT2 overexpression also promoted LD enwrapping (S5 Fig). Note that in *Caenorhabditis elegans*, DGAT2 is incorporated within membrane contact sites between ER and LDs [61], but to our knowledge, no LD-ER tethering function has been reported in mammalian cells. This LD-wrapping phenotype is unlikely to be antiviral *per se*, since it is also induced by HCV infection [30] and with the non-functional DGAT2 mutant (S5 Fig), however it suggests in both cases that intensified lipid exchanges take place between the organelles.

Consistent with these membrane reshuffling events, shifts both at the lipid class and lipid species levels have been observed upon HCV infection. In particular, the role of PI4P [17,62], cholesterol [21,25], SM [18], CER [51], PC [63], PE [20,51] and PA [22] lipids has been reported or discussed by others. We first investigated the localization of DAG, the substrate of DGAT2, by utilizing a previously described DAG lipid sensor [50]. Remarkably, while the DAG sensor localized in a Golgi-like and reticular pattern in the control cells, it accumulated near LDs in the DGAT2 cells. Some of the LDs were even fully enclosed by the sensor (Fig 7A and 7B). Consistently, due to its intrinsic negative curvature, the concentration of DAG in the ER was shown, at least in yeasts, to control the emergence of ER-embedded LDs and their budding [64]. However, we also observed an increased accumulation of the DAG-sensor at LD sites in the HuH6 cells (Fig 7H–7K). Since these cells were resistant to the antiviral effect of DGAT2 expression (Fig 7D), DAG mislocalization is unlikely the main driver of DGAT2 antiviral phenotype.

In our study, we could verify the upregulation of PC, PE and CER lipid classes upon HCV infection (Fig 8D). Furthermore, we also observed an increase of PUFA and oleoyl (C18:1) fatty acyl chain-containing PC and PE lipid species following HCV infection (Fig 9C and 9D), which had been described earlier [20,65,66]. The lipidomic changes observed in this study after HCV infection are therefore in agreement with the results of previous publications.

Surprisingly, we found that many of the DGAT2 overexpression-mediated lipid changes resembled those in HCV-infected cells (Figs 8 and 9). Altogether, DGAT2 overexpression also led to an increase in membrane lipids (Fig 8C), which are likely necessary to package the newly formed or larger LDs. Although no individual membrane lipid class was significantly increased upon DGAT2 overexpression, PC, PE and CER were also upregulated. Furthermore, we found that DGAT2 upregulated several PUFA-phospholipids (PUFA-PLs) while OA had opposite effects, suggesting this regulation might contribute to DGAT2 antiviral effect (Fig 9). This effect has to our knowledge not been reported so far but the enrichment of arachidonoyl (AA, C20:4) and alpha-linoleoyl (ALA, C18:3) PUFA in PLs was shown to be important for LD biogenesis [67–69]. The length and saturation of PL fatty acyl chains can influence the lipid bilayer fluidity and incorporation of long-chain and highly unsaturated PL generally leads to higher membrane flexibility and curvature [52]. In summary, both HCV infection and DGAT2 overexpression seem to upregulate lipid classes and species that increase membrane curvature and fluidity. Building on these results, we therefore hypothesize that due to the mutual need of membrane curvature-shaping lipids in LD packaging and DMV genesis, DGAT2-mediated LD biogenesis and HCV membranous web formation compete for similar host cell lipid pools. Curiously, this only applies to DGAT2-mediated LD biogenesis and not to OA-induced LD formation (Figs 8 and 9) and we therefore sought to uncover the reason behind this DGAT2-specific effect. It has been reported, that DGAT2 preferentially catalyzes lipids of the *de novo* lipogenesis pathway, including lipids with C12-C14 as well as (C16:0), (C16:1), (C18:0) and (C18:1) acyl chains [70,71]. Consistently, we detected a strong DGAT2-mediated depletion of one of the most abundant PC species, PC [18:1/18:1], which on the other hand, is strongly upregulated in HCV infected cells (Fig 9B and 9C). Also, other PLs with (C18:1) fatty acyl chains are depleted by DGAT2 overexpression (Fig 9C). Besides, we found a DGAT2-specific depletion but HCV-mediated increase of PE [18:1/22:6] (Fig 9B and 9D). The importance of *de novo* formed lipids and especially oleyl lipids for the HCV replication organelle has been documented [20,72] and the membrane bending properties of docosahexaenoyl (DHA, C22:6) PL [73] as well as its role in the formation of lipid rafts [74] are most likely beneficial for the HCV replication organelle formation. Given that these lipids are downregulated by DGAT2 but not by OA treatment, we speculate that DGAT2-mediated antiviral effect partially also derives from the DGAT2-specific substrate channeling.

**Fig 10:**
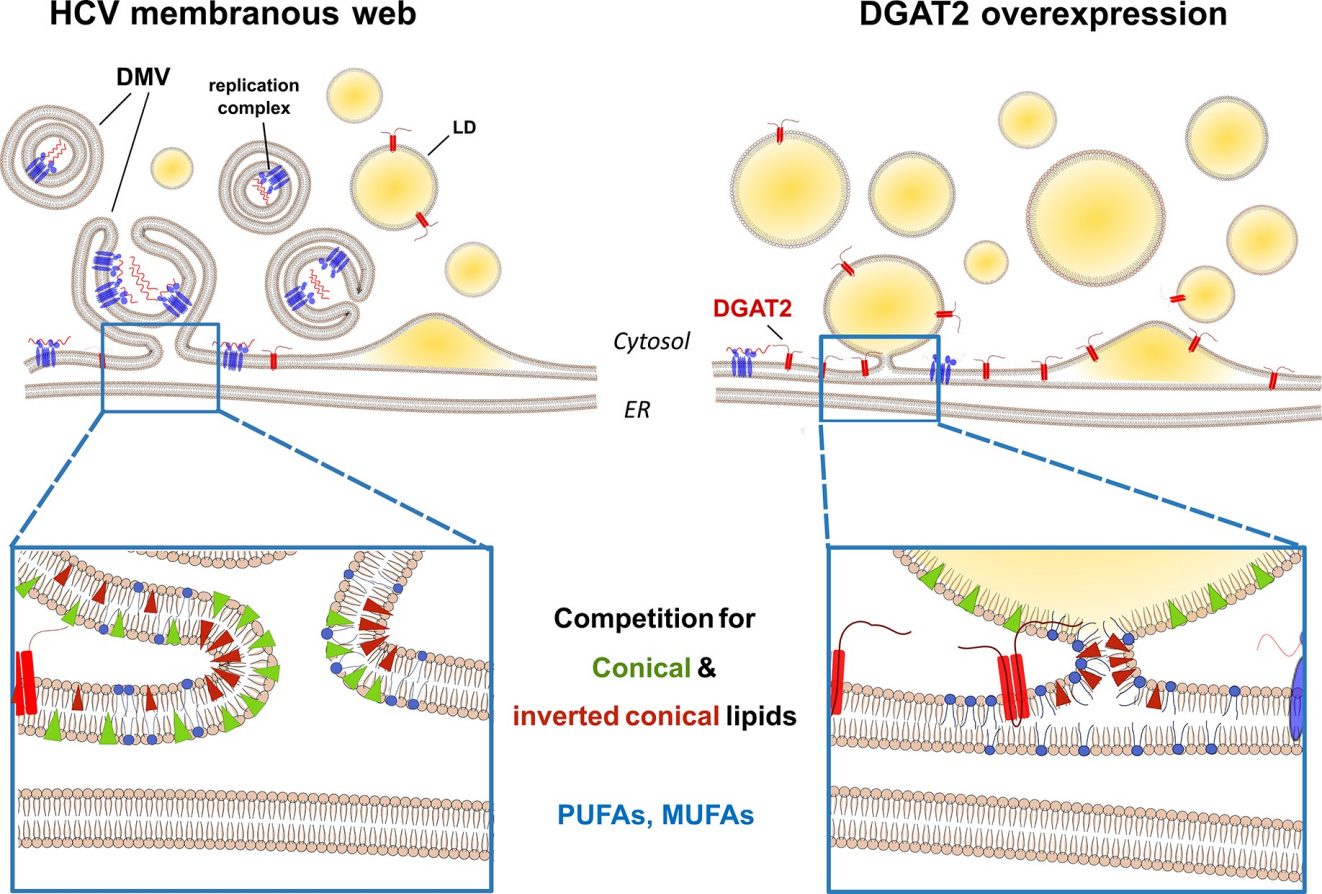
Model proposing overlapping lipid requirements for HCV DMV synthesis and LD biogenesis upon increased DGAT2 expression. Both HCV membranous web (left panel) and LD biogenesis (right panel) reshuffle the host cell lipid landscape to favor higher membrane curvature and flexibility and therefore accumulate conical and inverted-conical lipids as well as PUFAs and MUFAs, as described in the main text. Due to the excessive LD biogenesis and DGAT2 specific substrate preferences, we hypothesize that lipids crucial for the DMV synthesis are channeled towards LD expansion sites upon DGAT2 overexpression and their amount becomes limiting for the establishment of the HCV replication organelle. HCV replication factories are represented as blue protein complexes embedded in the ER / DMVs, the replicating genomes are depicted as red wavy lines and DGAT2 is shown as a red membrane-spanning protein. Conical and inverted conical lipids are shown in green and red triangles, respectively. MUFA and PUFA phospholipids are depicted in blue.

Altogether, we propose a model, in which the excessive LD formation mediated by DGAT2 expression leads to an unfavorable environment for DMV formation due to (i) competition for membrane curvature-inducing lipids and (ii) substrate channeling into TAG leading to the depletion of specific lipids required for the DMV biogenesis. These depleted lipids are essential either because they confer specific physical properties to the ER membrane or for their capacity to associate with specific HCV replication cofactors (Fig 10).

Finally, while DGAT2 overexpression system is artificial and we do not claim that DGAT2 actually restricts HCV replication *in vivo*, our experimental setup highlights a new link between LD biogenesis and the formation of HCV replication organelle. Furthermore, the effects we report here globally on the lipid landscape of the cells in presence of DGAT2 overexpression might be relevant locally, at the scale of specific nanodomains of the ER, in a more physiological context. Indeed, there is accumulating evidence that the intracellular membranes are not homogenous in terms of lipid composition but that individual organelles consist of micro- or nanodomains with specific lipid and protein content and with distinct functions [52,75]. Thus, despite being mostly disordered, the ER contains functional lipid nanodomains such as liquid-ordered lipid rafts and solid-ordered / gel-like domains that can serve as diffusion barriers [76]. In particular, membranes at membrane-contact sites are characterized with distinct physical properties and seem to have a unique lipidome, the latter of which is likely a signature of the lipids channeled between the connected organelles [77–79]. Therefore, we propose that LD biogenesis and the accompanying lipid flux between ER and LDs might locally affect the properties of the ER membrane and participate in determining the sites for efficient membrane reshuffling and viral replication organelle formation. Combined with the participation of lipid droplets in HCV morphogenesis and the required proximity between replication and assembly factories, membrane contact sites and inter-organelle lipid flux likely contribute to a very precise spatial compartmentalization of HCV infection, at the suborganellar level, both within the ER and among the different LDs of the cells. The recent advances in super resolution microscopy and imaging of lipids in live cells will be instrumental to put this model to the test.

## Materials and methods

### Ethics statement

All tissue donors gave written informed consent for experimental use of clinical data and liver specimen prior to surgery. The protocol was approved by the ethics commission of Hanover Medical School (#252–2008 and #2148–2014).

### Plasmids

All plasmids used in this study are listed in S1 Table. For this study, we ordered the human DGAT2 sequence as a gBlock (Integrated DNA Technologies, IDT) and cloned it between the AscI and SpeI restriction sites in the pWPI-Puro vector. We cloned the HA-tagged DGAT2 by insertion of a double HA tag (YPYDVPDYA, twice) preceded by a linker (GGGGSG) by PCR. Furthermore, we generated the point mutants DGAT2_L83A, DGAT2_HPH161-163AAA by fusion PCR. For the panel of further DGAT2 mutants, we split the DGAT2 sequence in three fragments by PCR amplification (N-terminal, middle part, C-terminal) and ordered gBlock (IDT) carrying the desired deletions within the respective fragment. The fragments were inserted into the pWPI_Puro vector by 3-insert-Gibson assembly (Gibson Assembly Master Mix, New England BioLabs (NEB) #E2611L) to generate pWPI_HAHA-L-DGAT2-del30-67_Puro, pWPI_HAHA-L-DGAT2-del66-115_Puro, pWPI_HAHA-L-DGAT2-mito4A_Puro, pWPI_HAHA-L-DGAT2-del327-350_Puro and pWPI_HAHA-L-DGAT2-insert-HA_Puro. We generated pLenti_CMV-TO_HAHA-DGAT2_Puro by restriction cloning into the pLenti_CMV-TO_Puro_DEST backbone. The DAG-sensor pWPI_mRuby3_PKCe_C1a_C1b_Puro was cloned as described in [50]. We ordered the sequence of the human protein kinase C epsilon (PKCe) (NM_005400.3) C1a-C1b (amino acids 170–294) cassette fused with mRuby3 as gBlock (IDT) and inserted it between the AscI and SpeI sites in the pWPI_Puro vector. We introduced the point mutation W264G [50,80] by fusion PCR. All cloned constructs were verified by multiple restriction analyses and by sequencing (Microsynth Seqlab). Primer sequences and detailed cloning procedures are available upon request.

### Antibodies and reagents

All antibodies used in this study are listed in S2 Table. Cell culture reagents and chemicals are detailed in S3 Table, except enzymes and kits which are given in the text. We dissolved and stored DGAT2i (PF-06424439) as 10 mM stock in DMSO. For lipid droplet induction, we mixed oleic acid (OA) and bovine serum albumin as described before [28] with the final concentration of OA indicated in the respective figure. We dissolved Daclatasvir, Doxycycline (Dox) and Cycloheximide (CHX) in DMSO as 10 mM stock solutions before further dilution. We dissolved BODIPY 493/503 as 1 mg/mL stock in DMSO. We prepared DAPI as 5 mg/mL stock in water and LD540 as 2.5 mg/mL stock in ethanol.

### Cell and virus culture

In this study, we used Lunet N hCD81 [81], Lunet N hCD81 FLuc [28], Lunet N hCD81/mRuby2 [29], Huh-7.5 [82], Huh-7.5.1 [83], HuH6 [84], HEK 293T [85], A549 [86] and Vero E6 [87] cells and cultured them using the previously described conditions. Note that the Lunet N hCD81 cells are a human hepatoma cell line derived from Huh7 cells and are highly permissive for HCV RNA replication [88]. We generated the Lunet N hCD81 [empty], [DGAT2], [DGAT2_L83A], [DGAT2_HPH161-163AAA] and [HA-DGAT2] cell lines by lentiviral transduction of the according constructs and puromycin selection (2.5 μg/mL). We generated the Dox-inducible cell line Lunet N hCD81/TetR [HA-DGAT2] by lentiviral transduction of pWPI_TetR_BLR (and selection with 5 μg/mL blasticidin for three passages) followed by transduction of the pLenti_CMV-TO_HAHA-DGAT2_Puro expression vector and Puromycin selection (as above).

All cell lines were cultivated at 37°C with 5% CO_2_ in Dulbecco’s modified Eagle’s medium (DMEM) supplemented with 2 mM L-glutamine, non-essential amino acids, 100 U/mL penicillin, 100 μg/mL streptomycin and 10% fetal bovine serum (FBS). In addition to this, we kept the cell lines under the relevant selection. We cultivated all Lunet N hCD81 cell lines in presence of blasticidin (5 μg/mL) to maintain CD81 expression. Additionally, Lunet N hCD81/mRuby2 and the newly generated Lunet N hCD81 DGAT2 cell lines were supplemented with puromycin (as above). The 8 PHH samples used in this study were the same as previously reported and therefore were isolated following the same procedure [29].

We used Huh-7.5 or Huh-7.5.1 cell lines for HCV Jc1 and JcR2a virus production and HEK 293T cells for lentivirus production following previously described protocols [28]. We used Vero E6 cells to amplify ZIKV (strain H/PF/2013, kindly provided by the Centre National de Référence des arbovirus IRBA at the Aix-Marseille University, France, and distributed via the European Virus Archive Global, EVAg Ref-SKU:001v-EVA1545) and LGTV (strain TP21, a kind gift from Gerhard Dobler) virus stocks. HCoV 229E RLuc was kindly provided by Volker Thiel and amplified on Huh-7.5 cells. Stocks of HEV Kernow-C1 p6 were produced as described before [89].

### *In vitro* transcription

HCV *in vitro* transcripts (IVTs) of pFK_i389_JcR2a_dg_Jc1 (JcR2a) [17], pFK_JFH1/J6/XbaI/C-846_dg (Jc1) [90], dbn3acc-sgr-cpg-low-luc2-ns5ac (SGR-DBN3A, a kind gift from Mark Harris) [91], pFK_i389LucNS3-3´’_JFH_dg.gb (SGR-JFH1) [92] and pFKi_341_PiLuc_NS3-3´_Con1 ET (SGR-Con1) [93] were generated. In brief, we linearized the plasmids with MluI or XbaI (for SGR-DBN3A) or SpeI (SGR-Con1). Digested plasmids were extracted using the Qiaquick Spin mini prep kit (Qiagen #27106) and dissolved in RNAse-free water. *In vitro* transcription mixtures comprised 80 mM HEPES [pH7.5], 12 mM MgCl2, 2 mM spermidine, 40 mM dithiothreitol (DTT), each nucleotide triphosphate at a concentration of 3.125 mM, 1 U RNasin/μL (NEB #M0314) of reaction volume, 0.2 μg restricted plasmid DNA/μL, and 0.6 U T7 RNA polymerase/μL (NEB #M0251). We incubated the reactions for 2 h at 37°C and added additional 0.3 U T7 RNA polymerase/μL to the mixture following another 2 h incubation. We terminated the transcription by addition of 3.25 U of RNase free DNase (NEB #M0303) per μg of plasmid DNA and incubation for 30 min at 37°C. We extracted the RNAs using the NucleoSpin RNA Clean-up Kit (Macherey-Nagel #740948.250), eluted them in RNAse free water and stored them at—80°C after concentration determination with a spectrophotometer (Implen). pIRF1b [38] IVTs were generated as described above but with an additional capping step during the *in vitro* transcription using the Ribo m7G Cap Analog (Promega #P1711).

### Luciferase activity assays

For *Renilla* (RLuc) and Firefly (FLuc) luciferase measurements we lysed the cells in Milli-Q water (400 μL per well for 6-well dishes, 150 μL per well for 12- or 24-well dishes, 40 or 50 μL per well for 96-well dishes) and froze the plates at—20°C or—80°C until luciferase readout. We performed the assays by following previously published protocols [94].

### HEV replication and focus forming assay

For HEV progeny particle production in stable Lunet N hCD81 cell lines we seeded 5x10^4^ cells per well in a 24-well plate, followed by infection with non-enveloped HEV Kernow-C1 p6 at a multiplicity of infection of 0.5. We removed the supernatant 24 hpi and added fresh culture medium. We trypsinized the cells 3 days post infection, neutralized with complete DMEM and centrifuged for 5 min at 500 g. After resuspension of the cell pellet in 100 μL cell culture media, we harvested intracellular progeny virus by lysing the cells via three freeze-thaw cycles. To remove cell debris, we centrifuged the lysate for 10 min at 10,000 g. For quantification of recovered HEV particles, we titrated the supernatant in duplicates on HepG2/C3A cells, which we seeded 24 h before titration with 10^4^ cells per well on 96-well plates. We fixed the cells 7 days post infection with 3% PFA and permeabilized with 0.5% TritonX-100 as described below. To count the focus-forming units per well, we performed immunofluorescence staining using an HEV ORF2-specific polyclonal rabbit hyperimmune serum (kindly provided by R. Ulrich, Friedrich Loeffler Institute, Germany) targeting the HEV capsid protein followed by staining with a secondary antibody (goat anti-rabbit IgG, AlexaFluor (AF) 488) and DAPI (0.5 μg/mL).

### HCV whole replication cycle

To assess the whole replication cycle of HCV, we seeded 5x10^4^ cells of the stable Lunet N hCD81 cell lines in 12-well plates and infected the cells on the next day with 350 μL HCV JcR2a virus stock. We changed the medium 4 hpi. We harvested the cell lysates 48 hpi for luciferase readout (producers) and transferred the supernatant onto Huh-7.5 target cells in triplicates in 24-well plates. We harvested the cell lysates of target cells at 72 hpi.

### Translation reporter assay

For the translation reporter assay, we seeded Lunet N hCD81 cell lines in 96-well plates (10^4^ cells per well). On the next day, we changed the medium with 20 μM CHX or DMSO control 1 h before transfection with IVTs obtained from the pIRF1b construct (a kind gift from Jean Dubuisson and Annie Cahour). We lysed the cells 8 hpt for FLuc and RLuc luciferase readout.

### JcR2a replication kinetics in Lunet N hCD81/TetR [HA-DGAT2] cells

We seeded 2x10^4^ Lunet N hCD81/TetR [HA-DGAT2] and [empty] cells in 24-well plates and infected the cells with JcR2a virus on day 2. We changed the medium of the infected cells 4 hpi and harvested the cell lysates for luciferase readout at 24, 32, 40 and 48 hpi. We induced HA-DGAT2 expression in the Lunet N hCD81/TetR [HA-DGAT2] cells 24 hours before infection or 4 or 24 hpi by 10 μM Dox treatment.

### Immunofluorescence

#### NS5A puncta quantification

We electroporated 4x10^6^ cells of the stable Lunet N hCD81 DGAT2 cell lines with 2 μg JcR2a IVTs and transferred the transfected cells on coverslips in 24-well plates. We changed the medium 4 hpt and fixed the cells 48 hpt for 10 min with 3% PFA. We permeabilized the cells with 0.5% TritonX-100 for 5 min and stained NS5A (anti-NS5A 9E10 followed by anti-mouse AF647 secondary antibody) and nuclei (DAPI, as above). We selected random fields showing NS5A-positive cells and acquired 10 images per coverslip as z-stacks with 60x magnification.

#### NS5A and HA-DGAT2 localization

We seeded 2x10^4^ Lunet N hCD81/ TetR [HA-DGAT2] cells on coverslips in 24-well plates and infected the cells with Jc1 on the next day. We performed a medium change at 4 hpi and either added 10 μM Dox or DMSO vehicle control to the cells. At 48 hpi, we fixed the cells with 3% PFA. We permeabilized the cells with 0.5% TritonX-100 for 5 min and stained NS5A (anti-NS5A 9E10 followed by anti-mouse AF488 secondary antibody), HA-DGAT2 (anti-HA followed by anti-goat AF647 secondary antibody) and nuclei (DAPI, as above).

#### LD quantification

For the LD quantification (Fig 4), we co-seeded 2.5x10^4^ cells of the stable Lunet N hCD81 DGAT2 cell lines with 2.5x10^4^ Lunet N hCD81/mRuby2 cells on coverslips in 24-well plates. Two days later, when the cells reached 70–80% confluence, we fixed the cells for 10 min with 3% PFA. We stained the cells with BODIPY 493/503 (1 μg/mL) and DAPI (as above). We acquired 10 images per coverslip at random positions with 60x magnification.

#### HA-DGAT2 co-staining with organelle and lipid markers

We seeded 2x10^4^ Lunet N hCD81 or Lunet N hCD81/FLuc cells on coverslips in 24-well plates. On the next day, we transduced the cells with the DGAT2 expression constructs and changed the medium 6 hours post transduction. On day 3, we triggered LD accumulation by 360 μM OA treatment overnight (see above). On the next morning, we fixed the cells for 10 min with 3% PFA. We permeabilized the cells for 5 min with 0.5% TritonX-100 before immunofluorescence staining. We first stained HA-DGAT2 with anti-HA (goat) and anti-goat-AF647 before co-staining with either anti-Calnexin (rabbit, anti-rabbit-AF488), anti-ADRP (mouse, anti-mouse-AF488), anti-CoxIV (mouse, anti-mouse-AF488), BODIPY 493/503 or LD540 (a kind gift of Christoph Thiele) [95]. Cell nuclei were visualized with DAPI.

#### DAG localization

We seeded 2x10^4^ Lunet N hCD81 and 2.5x10^4^ HuH6 cells on coverslips in 24-well plates in the morning. We transduced the cells 4 h later with the DAG sensor expression construct (Fig 7) or the mutant version (S8 Fig) combined with either HA-DGAT2 or the empty control construct. We changed the medium 4 h post transduction. On day 4, we fixed the cells for 10 min with 3% PFA. We permeabilized the cells for 5 min with 0.5% TritonX-100 and stained HA-DGAT2 with anti-HA and anti-goat-AF647 antibodies. We visualized the LD and nuclei by BODIPY 493/503 or DAPI staining.

### Image acquisition and analysis

Unless otherwise stated, the images were taken with a 60x Nikon CFI Apochromat TIRF objective (CFI Apochromat TIRF 60x Oil/ 1.49/ 0,13). We acquired the fluorescence microscopy images for LD and NS5A quantification with a Nikon Ti-E microscope equipped with a Yokogawa CSU-X1 spinning disc and an EMCCD DU-888 camera from Andor and an additional 2x magnification lens inside the spinning-disc unit. We acquired the NS5A and HA-DGAT2 localization images with a Nikon AX microscope equipped with a 60x oil objective and a DUX-VB4 DUVB 4Ch detector and using the Galvano scanning mode. All other images were taken with a Nikon Ti2 microscope equipped with an Ai plus laser scan confocal unit.

We prepared the depicted images in Fiji [96] with equal background subtraction and contrast enhancement parameters for all samples of a figure panel. We measured intensity profiles along the indicated lines in Fiji, normalized intensities to the maximal intensity of the fluorochrome along the given line and plotted them with GraphPad Prism.

NS5A puncta were quantified using a self-written Fiji macro. We first automatically segmented nuclei and NS5A puncta in all z-slices of the pictures using the same parameters throughout all conditions. We then filtered out duplicate NS5A puncta of consecutive slices of the z-stack to obtain the total number of NS5A puncta per field of view. We measured the perinuclear NS5A signal by dilating the area of the segmented nuclei, which allowed us to identify HCV-positive cells and to calculate their total nuclear area per field of view—a convenient surrogate for the total cellular area of the infected cells. With this, we could normalize the number of NS5A puncta for the nuclear area of HCV-positive cells for each field of view. Finally, we obtained the puncta density by multiplying this value with an arbitrary average nuclear area of 30,000 pixels.

For the LD quantification, we first automatically segmented mRuby2-positive cells with a Fiji macro to generate binary masks in order to distinguish mRuby2-positive and negative cells. Nuclei and LDs of both populations were then automatically segmented. Images with successful segmentation were further analyzed with CellProfiler [97] using the same parameters throughout all conditions.

### Plaque forming unit assay

For ZIKV and LGTV plaque assays, 2.5x10^4^ Vero E6 or A549 cells per well were seeded in 96-well plates. On the next day, we applied the infectious supernatants in serial dilution to the cells. Two hours after infection, we replaced the medium with overlay medium (DMEM with 1.5% CMC and 5% FBS for ZIKV, DMEM with 0.75% CMC and 1% FBS for LGTV) and incubated the cells for 96 h. We fixed the cells with 10% formaldehyde and visualized the plaques by crystal violet staining (1% crystal violet, 10% ethanol) and counted them manually.

### CLEM and DMV quantification

For the DMV characterization by CLEM microscopy, we transfected Huh7-Lunet/T7 [empty], [DGAT2], or [DGAT2_HPH161-163AAA] cells with HCV NS3-5B/5A^EGFP^expression plasmid by lipofectamine transfection according to the manufacturer protocol. After 24 h, we identified transfected cells by GFP signal (wide field fluorescence microscope) and fixed the cells for CLEM analysis. We acquired TEM images x4 k magnification and utilized systematic random sampling and placed ~100 μm^2^ rectangle areas on whole cell images to count and measure DMVs and LDs manually. We counted at least 10 GFP-positive cells in each sample (n ≥ 10).

### Flow cytometry-based quantification of the lipid droplet content

To quantitatively assess the LD content by flow cytometry we adapted our previously published protocol [29] with the following changes. For Figs 4B, 6E and S2 we transduced the cells in 12-well dishes on day 2 with the respective DGAT2 expression constructs or the empty vector control and changed the medium 4 h post transduction. We triggered LD accumulation in the dedicated samples by OA addition on day 3 overnight, with the indicated concentration (Fig 4B). Cell harvest, addition of reference cells, fixation and staining was performed as before [29] and analysis was done with the BD Accuri C6 flow cytometer (Fig 4A and 4B) or the BD LSR Fortessa Cell Analyzer (Figs 6E and S2).

For Fig 7E and 7F we followed the same protocol except that we seeded the cells in 6 cm cell culture dishes (10^5^ Lunet N hCD81, 2x10^5^ HuH6) and upon cell harvest kept two third of the cells for RT-qPCR before spiking in the reference cells. Samples were analyzed with the Sony Spectral Analyzer.

In all cases, we set gates to exclude cell debris by using the FSC-A and SSC-A channels as well as doublet cells using the FSC-A and FSC-H channels. We obtained BODIPY 493/503 and mRuby2 fluorescence signals by using the FL1 and FL3 (BD Accuri) or B2 and YG3 (BD Fortessa) channels, or the Spectral Analyzer (Sony) respectively.

We performed color compensation or spectral unmixing following the manufacturer´s instructions. We distinguished test cells from the spiked-in control cells by their red fluorescence as shown in Fig 4A and compared the ratios of the BODIPY fluorescence between the test and the control cells as explained in the main text. Further analysis was performed with FlowJo (BD Biosciences).

### RT-qPCR

To analyze the expression levels of DGAT2, RT-qPCR was performed. We extracted cellular RNA using the NucleoSpin RNA extraction kit (Macherey-Nagel #740955) according to the manufacturer’s instructions and assessed the quality of the extracted RNA by gel electrophoresis. RNA transcripts of GAPDH and DGAT2 were quantified by one-step primer-probe RT-qPCR using the LightCycler 480 RNA Master Hydrolysis Probes kit (Roche # 04991885001, Fig 1B) or the Luna Universal Probe One-Step RT-qPCR kit (NEB # E3006, Fig 7E) following the manufacturer’s instructions. Utilized primers and probes can be found in S4 and S5 Tables. Within one reaction, we combined primers and probes for GAPDH and DGAT2 and optimized the concentrations of all oligonucleotides within one reaction by titration. We performed four biological replicates of sample preparation and measured each sample in technical triplicates on a Lightcycler 480 instrument (Roche) or with the 7900HT Fast Real-Time PCR system (Applied Biosystems). We analyzed the data by the 2^- ΔΔCt^ method.

### Extraction of microsomal fractions

To analyze whether HA-DGAT2 and HA-DGAT2_del66-115 were present in the microsomal fractions, we seeded Lunet N hCD81 in 15 cm dishes to be confluent on day 4. We transduced the cells with lentiviruses to express empty, HA-DGAT2 or HA-DGAT2_del66-115 and changed the medium 6 hours later. When relevant, we treated the cells with 360 μM OA combined with BSA on day 3 overnight. On day 4, we harvested the cells by trypsinization and adjusted the cell number equally throughout the conditions. We washed the cells with PBS (500 g, 5 min) and resuspended them in sucrose-based medium (0.25 M sucrose, 10 mM Hepes, 1 cOmplete Mini Protease Inhibitor Cocktail tablet per 10 mL medium). We lysed the cells with a Dounce homogenizer on ice and controlled the cell rupture by Trypan Blue staining (same stroke number per cell type for all conditions). We removed the cell debris and nuclei from the lysate by centrifugation (2x 500 g, 5 min). For the microsome extraction, we centrifuged the remaining supernatant at 10,300 g for 10 min at 4°C to separate crude mitochondria. The procedure was repeated three times before transferring the remaining lysate into ultracentrifugation tubes filled with the sucrose-based medium. We centrifuged the samples at 100,000 g for 1 h at 4°C. Afterwards, we discarded the supernatants and resuspended the pellets containing the microsomal membranes in sucrose-based medium. We added Western blot sample buffer to both cytoplasmic and microsomal extracts and stored the samples at—20°C.

### Sample preparation for lipidomics

We seeded Lunet N hCD81 stable DGAT2 cell lines in 10 cm dishes to be confluent on day 4. When relevant, we infected the cells on day 2 with Jc1 and changed the medium 4 hpi or incubated the cells on day 3 overnight with 360 μM OA combined with BSA (as above). We harvested the cell pellets on day 4 (48 hpi) by trypsinization and counted them with a Neubauer chamber. We performed Dounce homogenization to lyse the cells as described above and separated the cell debris and nuclei from the homogenate by centrifugation (2x 500 g, 5 min). Cleared cell lysates were stored at—80°C until lipid extraction.

### Mass Spectrometry based lipidomic analysis

Lipidomic analysis was done with the Lipidyzer platform. This included a triple quadrupole mass spectrometer (QTRAP 5500; AB SCIEX, Darmstadt, Germany) equipped with a differential mobility spectrometer (DMS) interface operating with SelexION technology. In this case, an ultra-high pressure liquid chromatography system (Nexera X2, Shimadzu, Japan) was used as an autosampler. The Lipidyzer Platform was tuned using the SelexION Tuning Kit (AB SCIEX) and a system suitability test was performed using the System Suitability Kit (AB SCIEX) according to the manufacturer’s instructions. To the cell lysates, 100 μL of water was added and shortly vortexed. The lipid extraction process was then carried out as previously described [98]. The generated data was processed using Lipidomics workflow manager (AB SCIEX) and Shotgun Lipidomic Shortgun Assistant (SLA) software according to the SLA guidelines [99].

### MTT assay

To monitor growth and viability of the DGAT2-overexpressing cell lines, each cell line was seeded with 10^4^ cells per well in 96-well dishes and an MTT assay was performed at 4, 24, 48, 72 and 96 h post seeding. MTT was purchased from Sigma Aldrich (#M5655), dissolved in PBS at 5 mg/mL and this stock was diluted 10 times in complete DMEM before usage. At each time point, the cell supernatant was discarded, and the cells incubated with 50 μL per well of MTT-containing complete medium, previously prewarmed at 37°C. After 1 h incubation at 37°C, the medium was discarded, MTT precipitates were dissolved by adding 50 μL per well of DMSO, and absorbance was read at 570 nm in a Biotek Synergy 2 plate reader.

### Statistics

We depicted mean ± standard error of the mean (SEM) values in bar plots and mean ± standard deviation (SD) in all other plots, if not indicated differently. For statistical analysis we used GraphPadPrism, R [100] and RStudio [101]. We performed statistical analysis on absolute or log-transformed values using repeated-measured ANOVA test followed by Dunnet’s multiple comparison test in case of significant changes. For normalized data, we performed two-tailed unpaired t-test with Welch’s correction. Significant changes are indicated by asterisks in the respective figures and shown as *P < 0.05; **P < 0.01; ***P < 0.005; ****P < 0.001. We tested the lipidomics results by two-tailed students t-test with unequal variances and adjusted for multiple testing using the Benjamini-Hochberg procedure. We determined statistically significant values if the adjusted p-value (q-value) < 0.1.

## Supporting information

S1 Fig Effect of DGAT2 expression on cell viability. Cell viability of Lunet N hCD81 [empty], [DGAT2], [DGAT2_L83A] and [DGAT2_HPH161-163AAA] cells was measured by MTT assay at 4, 24, 48, 72 and 96 hours post seeding. Normalized values (to 4 h post seeding) are depicted (n = 3).
(TIF)

S2 Fig LD accumulation upon DGAT2 inducible expression. Doxycycline (Dox) inducible HA-DGAT2 Lunet N hCD81 cells were treated with Dox for 0, 8, 16, 24, 32, 40 or 48 h prior to harvest. Cells were mixed with a mRuby2-positive reference cell population and relative LD amount was measured by flow cytometry and calculated as described in the main text and in Fig 4A. Values were normalized to 0 h treatment and results of statistical tests are indicated by asterisks (n = 3–4).
(TIF)

S3 Fig HA-tagged DGAT2 limits JcR2a replication. Lunet N hCD81 cells were transduced with lentiviruses to express [empty], [DGAT2] or [HA-DGAT2]. Cells were infected with JcR2a 72 h post transduction. Cell lysates were harvested 4, 24, 48 and 96 h post infection and virus replication was measured by luciferase assay. Significant changes compared to the [empty] control group for each harvest time are shown. The asterisk color indicates the respective cell line (n = 3).
(TIF)

S4 Fig Effect of OA treatment on HCV replication. Lunet N hCD81 cells were seeded and infected with JcR2a on the next day. Medium change with either 0, 36, 100 or 360 μM OA supplemented with 30 μg/μL BSA was performed 4 h post infection. Cell lysates were harvested 48 h post infection and virus replication was measured by luciferase assay. Significant changes compared to the BSA-only treated control group are shown (n = 3).
(TIF)

S5 Fig Effect of DGAT2 expression on LD wrapping in HCV NS3-5B/5A^EGFP^expressing cells. Stable Lunet T7 cells overexpressing [empty] (upper panel) or [DGAT2] (lower panel) were transfected with the pTM expression vector encoding HCV NS3-5B/5A^EGFP^. Cells were fixed 24 hours post transfection. Transfected cells were first identified by their GFP signal then fixed and further processed for CLEM analysis. **(A)** Representative CLEM images. The yellow box area in the overview image (left panel) is enlarged in the right panel. Red stars indicate LDs enwrapped by ER membrane. Scale bar for overview image, 1 μm; for magnified image, 500 nm. **(B)** LD profiles were analyzed using TEM images taken at x4 k magnification and the percentage of ER-enwrapped LDs per image slice is depicted. Statistical tests were performed for the individual cell lines against the [empty] control group.
(TIF)

S6 Fig Effect of DGAT2 on other plus-strand RNA viruses. **(A)** Zika virus (ZIKV) and **(B)** Langat virus (LGTV) titers produced in Lunet N hCD81 cells expressing [empty], [DGAT2], [DGAT2_L83A] or [DGAT2_HPH161-163AAA], or Lunet N hCD81 cells expressing an shRNA against the ATP6VOC host factor (sh_ATP6VOC [102]) or a nontargeting shRNA (sh_NT). The cell lines were infected with ZIKV or LGTV at a multiplicity of infection of 0.1 for 96 h. Released infectious titers were measured by plaque assay and plotted as plaque forming units (PFU) per mL (n = 3–5). **(C)** Human coronavirus 229E (HCoV 229E) replication in stable DGAT2 Lunet N hCD81 cells. Cells were infected with HCoV 229E RLuc for 24 h and viral replication was measured by luciferase assay (n = 3). **(D)** Hepatitis E virus (HEV) progeny virus particles production in stable DGAT2 Lunet N hCD81 cell lines infected with HEV genotype 3 (Kernow-C1 p6 clone). Non-enveloped progeny virus was quantified by titration on naïve HepG2/C3A cells. Titers were determined by immunofluorescence microscopy and plotted as focus forming units (FFU) per mL (n = 4). Statistical tests were performed for the individual cell lines against the [empty] control group. Significant changes are indicated by asterisks.
(TIF)

S7 Fig Localization of HA-tagged DGAT2 mutants. Immunofluorescence microscopy localization studies of the localization deficient DGAT2 mutants completing the panels in Fig 6C and 6D. Staining and depiction is the same as in Figs 6C and 6D.
(TIF)

S8 Fig DAG sensor and sensor mutant localization in Lunet N hCD81 cells. We transduced the mRuby3-PKCe-C1a-C1b DAG sensor [50] **(A)** or its W264G mutant version **(B)** in Lunet N hCD81 [empty] cells. The DAG sensor is depicted in magenta. LDs were stained with BODIPY 493/503 (green) and nuclei with DAPI (blue). Note that contrast enhancement of both panels was performed according to the same percentage of saturated pixels to allow visualization of the weakly detected mutant DAG sensor. The white box area in the overview images (upper panel) is enlarged in the second row for each channel. Scale bar depicts 20 μm in the upper and 2 μm in the lower panels. Representative images of 3 independent experiments are shown.
(TIF)

S1 Table Plasmid constructs used in this study. (DOCX)

S2 Table Antibodies used in this study. (DOCX)

S3 Table Reagents and chemicals used in this study. (DOCX)

S4 Table RT-qPCR primers used in this study. (DOCX)

S5 Table RT-qPCR probes used in this study. (DOCX)
