# Supplementary figures and images for: The triglyceride-synthesizing enzyme diacylglycerol acyltransferase 2 modulates the formation of the hepatitis C virus replication organelle
Source: PLoS Pathog. 2024 Sep 6;20(9):e1012509. doi: 10.1371/journal.ppat.1012509 (PMC11410266; doi:10.1371/journal.ppat.1012509)

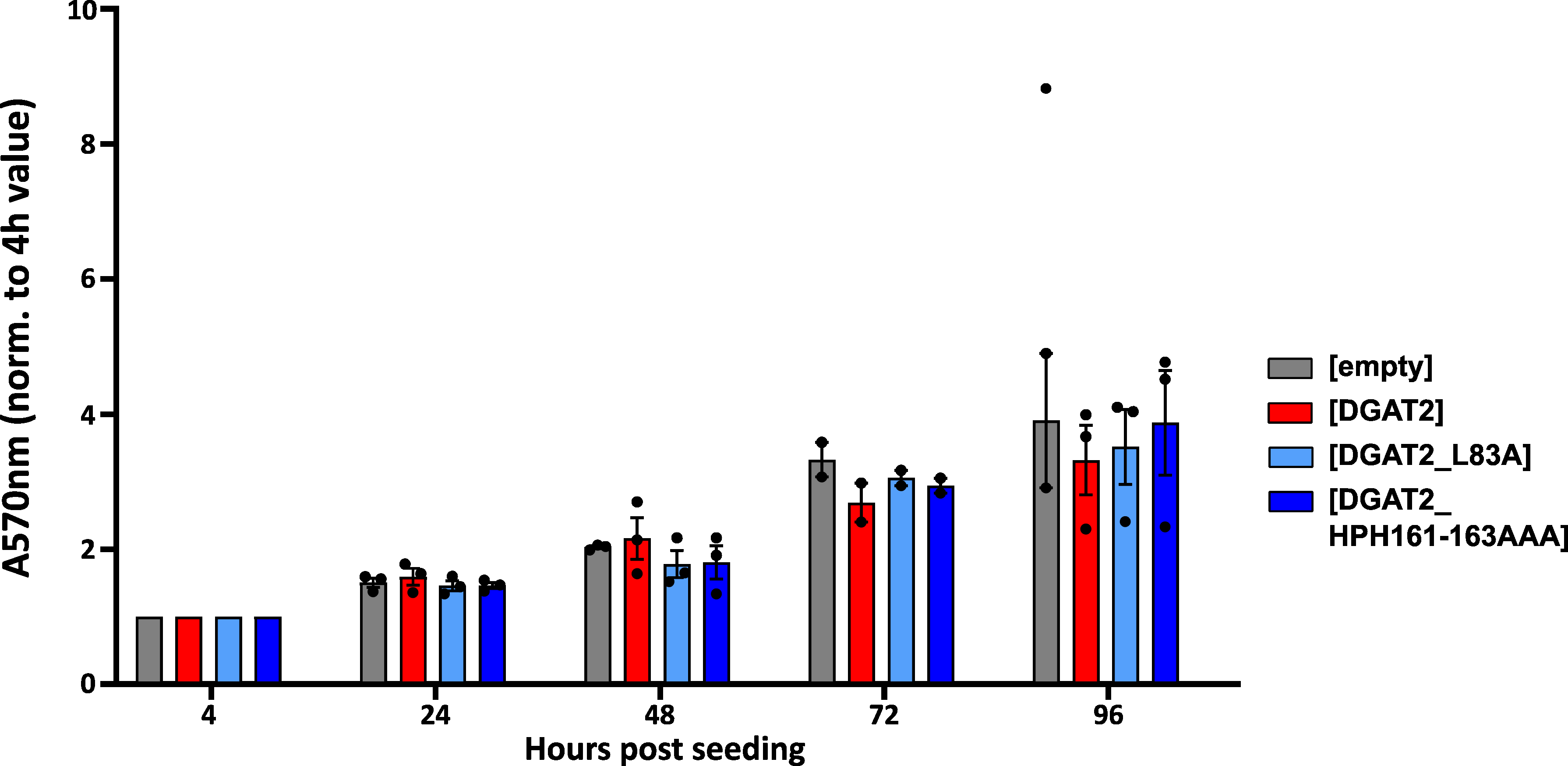

Supplement: S1 Fig — Cell viability of Lunet N hCD81 [empty], [DGAT2], [DGAT2_L83A] and [DGAT2_HPH161-163AAA] cells was measured by MTT assay at 4, 24, 48, 72 and 96 hours post seeding. Normalized values (to 4 h post seeding) are depicted (n = 3). (TIF) [file ppat.1012509.s001.tif]

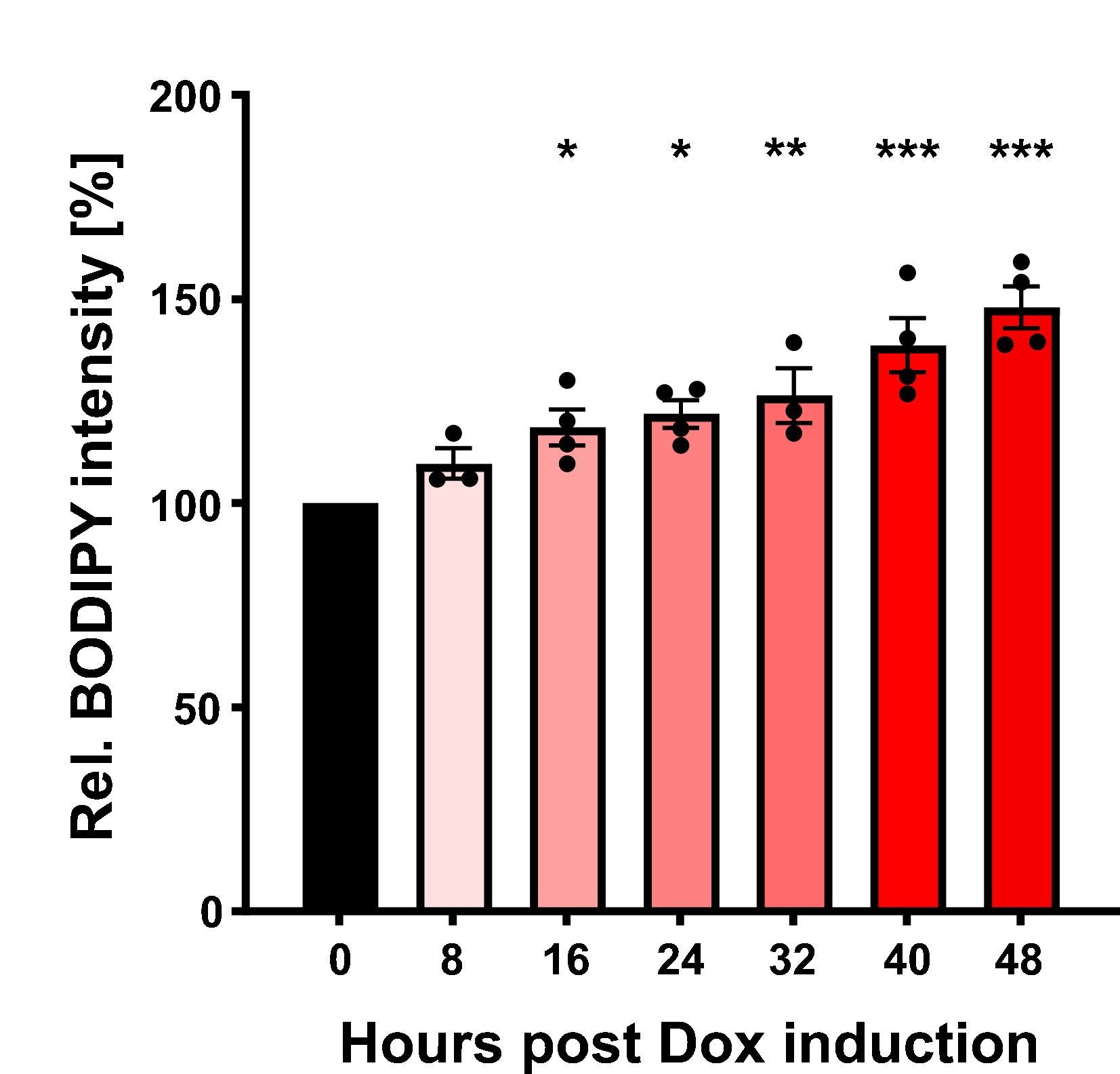

Supplement: S2 Fig — Doxycycline (Dox) inducible HA-DGAT2 Lunet N hCD81 cells were treated with Dox for 0, 8, 16, 24, 32, 40 or 48 h prior to harvest. Cells were mixed with a mRuby2-positive reference cell population and relative LD amount was measured by flow cytometry and calculated as described in the main text and in Fig 4A. Values were normalized to 0 h treatment and results of statistical tests are indicated by asterisks (n = 3–4). (TIF) [file ppat.1012509.s002.tif]

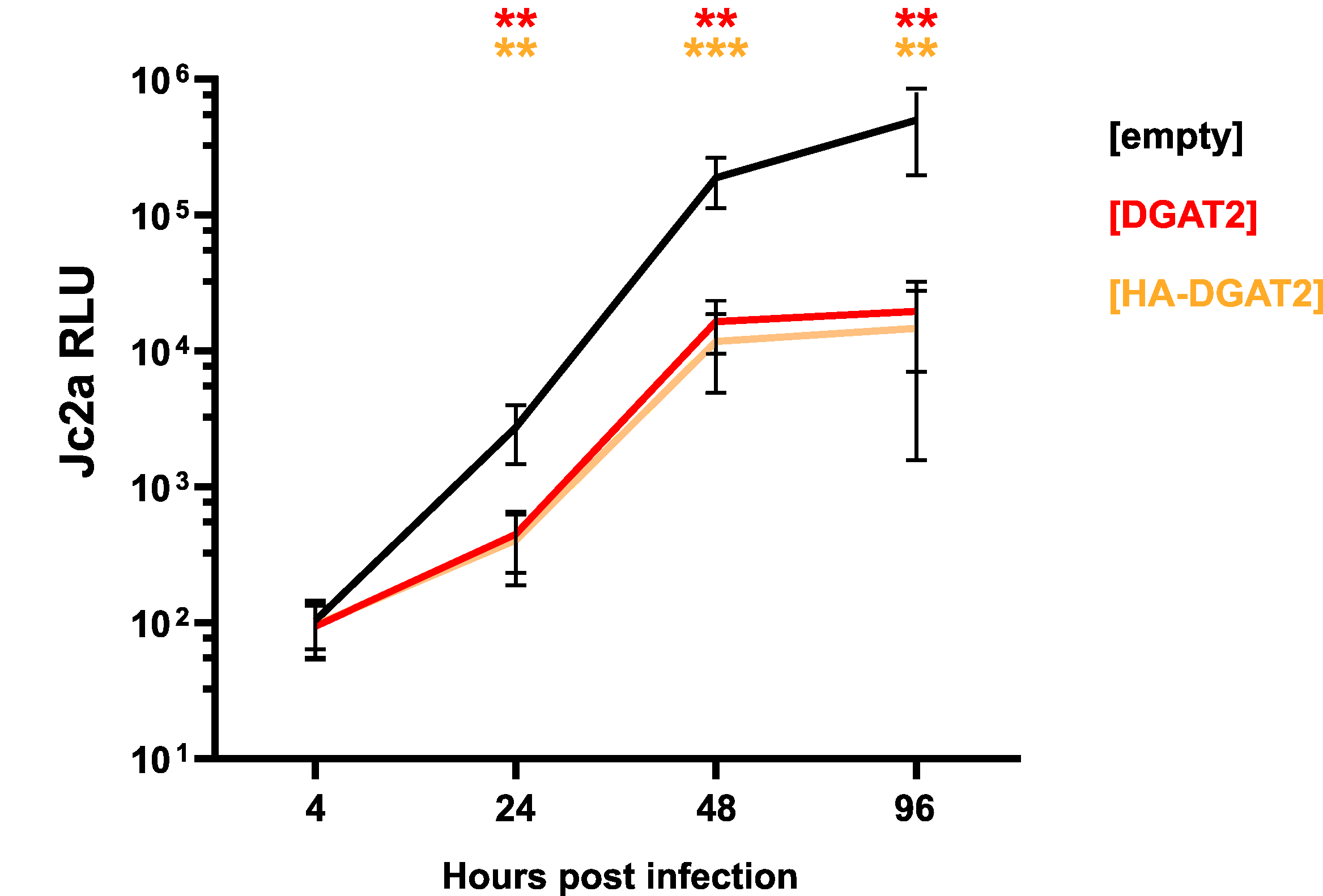

Supplement: S3 Fig — Lunet N hCD81 cells were transduced with lentiviruses to express [empty], [DGAT2] or [HA-DGAT2]. Cells were infected with JcR2a 72 h post transduction. Cell lysates were harvested 4, 24, 48 and 96 h post infection and virus replication was measured by luciferase assay. Significant changes compared to the [empty] control group for each harvest time are shown. The asterisk color indicates the respective cell line (n = 3). (TIF) [file ppat.1012509.s003.tif]

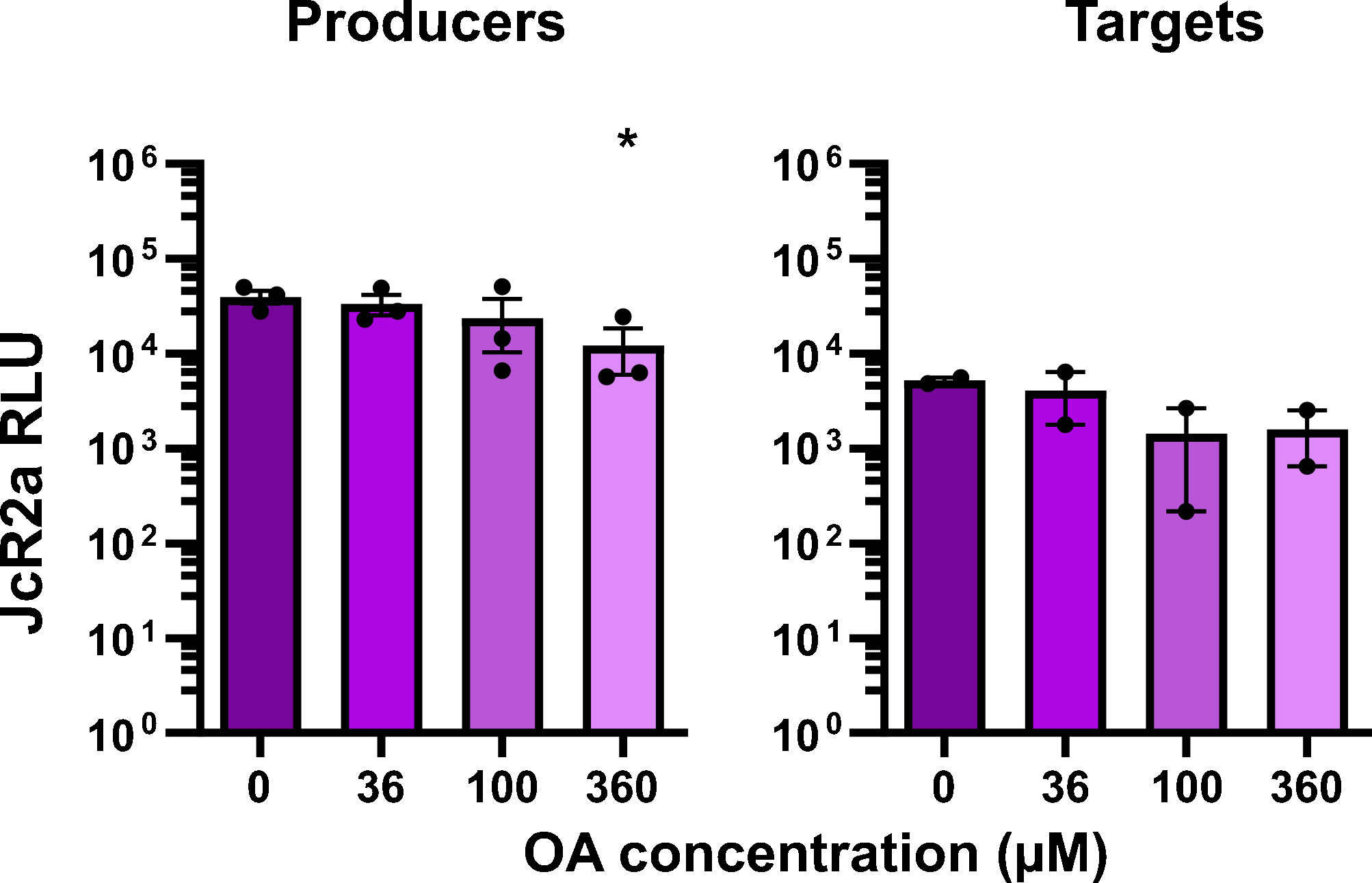

Supplement: S4 Fig — Lunet N hCD81 cells were seeded and infected with JcR2a on the next day. Medium change with either 0, 36, 100 or 360 μM OA supplemented with 30 μg/μL BSA was performed 4 h post infection. Cell lysates were harvested 48 h post infection and virus replication was measured by luciferase assay. Significant changes compared to the BSA-only treated control group are shown (n = 3). (TIF) [file ppat.1012509.s004.tif]

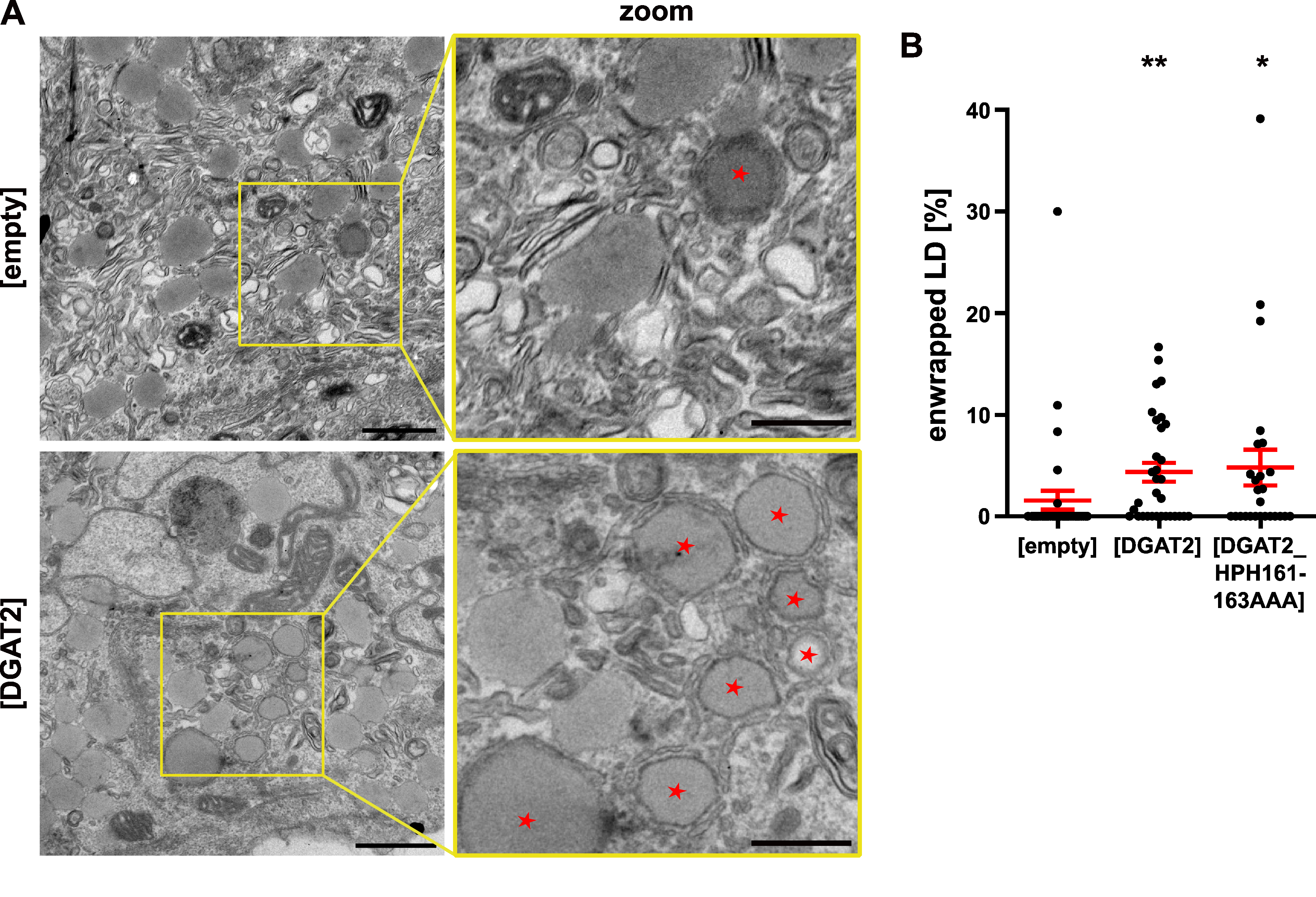

Supplement: S5 Fig — Stable Lunet T7 cells overexpressing [empty] (upper panel) or [DGAT2] (lower panel) were transfected with the pTM expression vector encoding HCV NS3-5B/5AEGFP. Cells were fixed 24 hours post transfection. Transfected cells were first identified by their GFP signal then fixed and further processed for CLEM analysis. (A) Representative CLEM images. The yellow box area in the overview image (left panel) is enlarged in the right panel. Red stars indicate LDs enwrapped by ER membrane. Scale bar for overview image, 1 μm; for magnified image, 500 nm. (B) LD profiles were analyzed using TEM images taken at x4 k magnification and the percentage of ER-enwrapped LDs per image slice is depicted. Statistical tests were performed for the individual cell lines against the [empty] control group. (TIF) [file ppat.1012509.s005.tif]

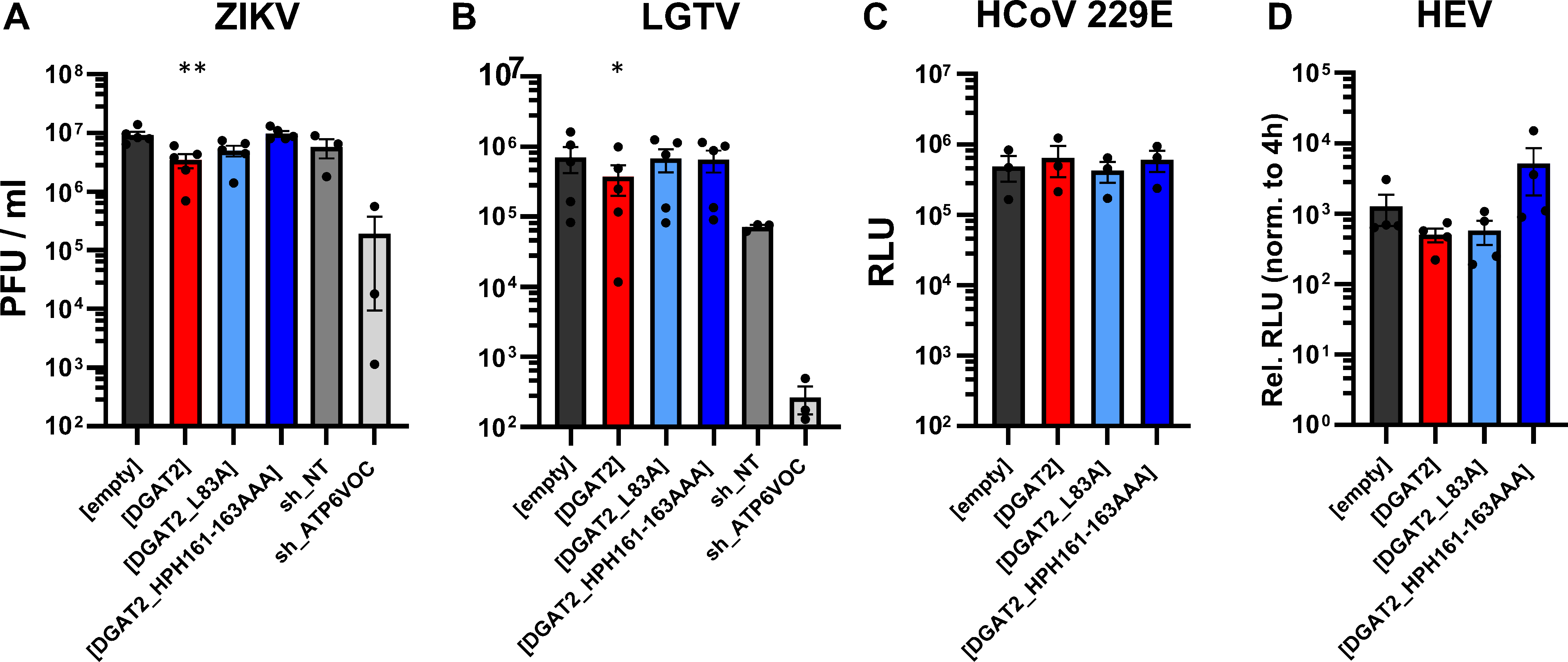

Supplement: S6 Fig — (A) Zika virus (ZIKV) and (B) Langat virus (LGTV) titers produced in Lunet N hCD81 cells expressing [empty], [DGAT2], [DGAT2_L83A] or [DGAT2_HPH161-163AAA], or Lunet N hCD81 cells expressing an shRNA against the ATP6VOC host factor (sh_ATP6VOC [102]) or a nontargeting shRNA (sh_NT). The cell lines were infected with ZIKV or LGTV at a multiplicity of infection of 0.1 for 96 h. Released infectious titers were measured by plaque assay and plotted as plaque forming units (PFU) per mL (n = 3–5). (C) Human coronavirus 229E (HCoV 229E) replication in stable DGAT2 Lunet N hCD81 cells. Cells were infected with HCoV 229E RLuc for 24 h and viral replication was measured by luciferase assay (n = 3). (D) Hepatitis E virus (HEV) progeny virus particles production in stable DGAT2 Lunet N hCD81 cell lines infected with HEV genotype 3 (Kernow-C1 p6 clone). Non-enveloped progeny virus was quantified by titration on naïve HepG2/C3A cells. Titers were determined by immunofluorescence microscopy and plotted as focus forming units (FFU) per mL (n = 4). Statistical tests were performed for the individual cell lines against the [empty] control group. Significant changes are indicated by asterisks. (TIF) [file ppat.1012509.s006.tif]

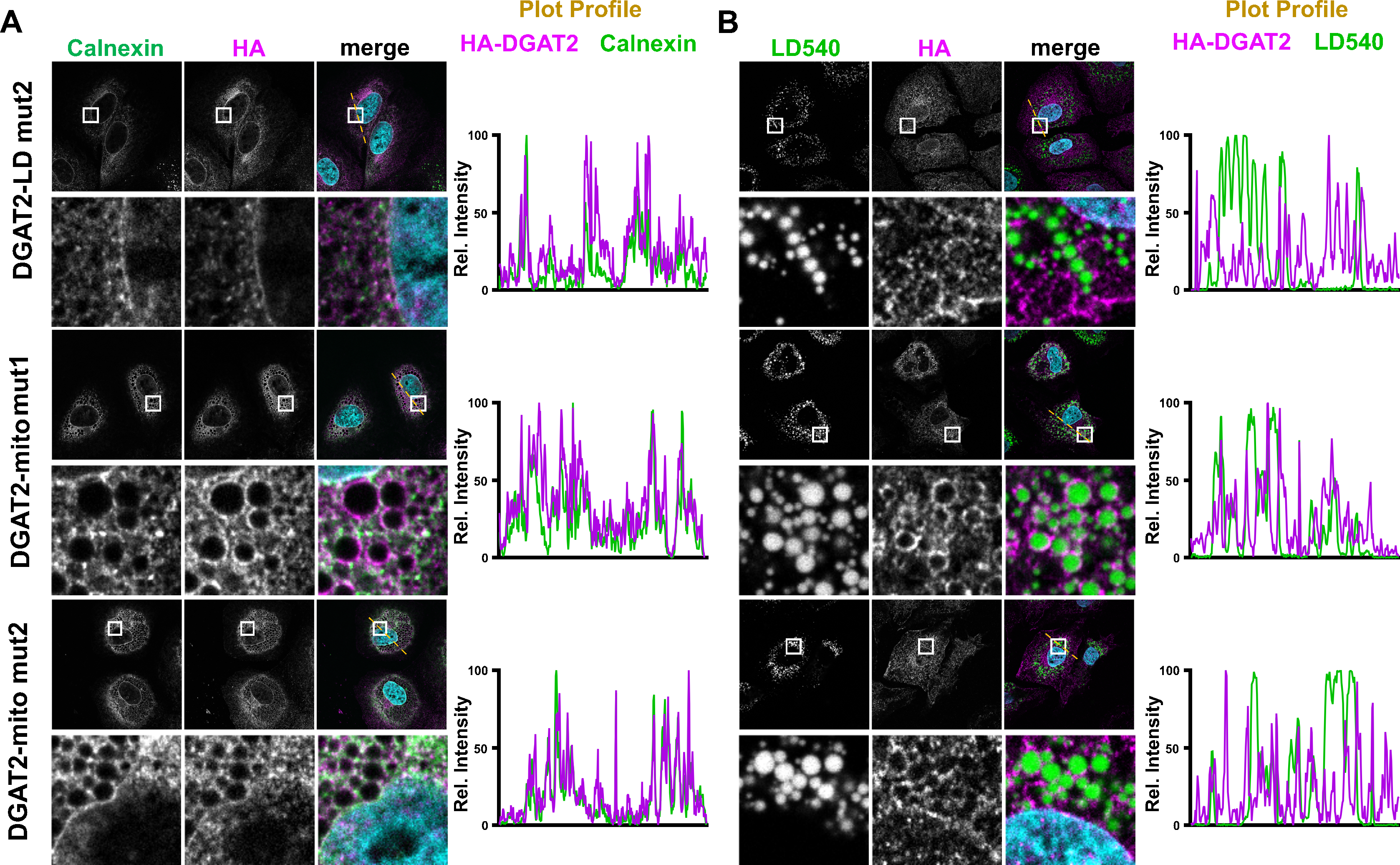

Supplement: S7 Fig — Immunofluorescence microscopy localization studies of the localization deficient DGAT2 mutants completing the panels in Fig 6C and 6D. Staining and depiction is the same as in Figs 6C and 6D. (TIF) [file ppat.1012509.s007.tif]

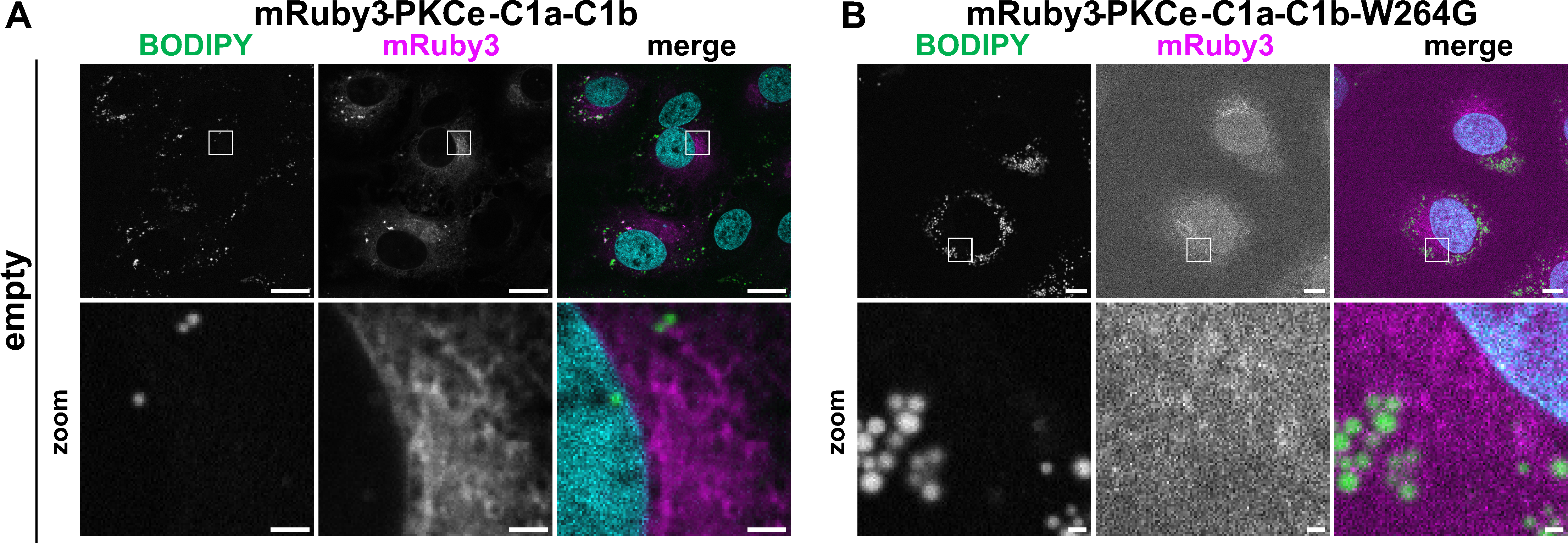

Supplement: S8 Fig — We transduced the mRuby3-PKCe-C1a-C1b DAG sensor [50] (A) or its W264G mutant version (B) in Lunet N hCD81 [empty] cells. The DAG sensor is depicted in magenta. LDs were stained with BODIPY 493/503 (green) and nuclei with DAPI (blue). Note that contrast enhancement of both panels was performed according to the same percentage of saturated pixels to allow visualization of the weakly detected mutant DAG sensor. The white box area in the overview images (upper panel) is enlarged in the second row for each channel. Scale bar depicts 20 μm in the upper and 2 μm in the lower panels. Representative images of 3 independent experiments are shown. (TIF) [file ppat.1012509.s008.tif]
