# Supplementary material for: The triglyceride-synthesizing enzyme diacylglycerol acyltransferase 2 modulates the formation of the hepatitis C virus replication organelle
Source: PLoS Pathog. 2024 Sep 6;20(9):e1012509. doi: 10.1371/journal.ppat.1012509 (PMC11410266; doi:10.1371/journal.ppat.1012509)
Supplement: S1 Table — (DOCX) [file ppat.1012509.s009.docx]

S1 Table: Plasmid constructs used in this study

| **Name** | **Reference** |
| --- | --- |
| pFK_i389_JcR2a_dg_Jc1 (JcR2a) | [1] |
| pFK_JFH1/J6/XbaI/C-846_dg (Jc1) | [2] |
| dbn3acc-sgr-cpg-low-luc2-ns5ac (SGR-DBN3A) | [3] |
| pFK_i389LucNS3-3'_JFH_dg.gb (SGR-JFH1) | [4] |
| pTM_NS3-3'_5A gfp 383_JFH.gb | [5] |
| pFKi_341_PiLuc_NS3-3´_Con1 ET | [6] |
| pIRF1b | [7] |
| pWPI_DGAT2_Puro | This study |
| pWPI_DGAT2_L83A_Puro | This study |
| pWPI_DGAT2_HPH161-163AAA_Puro | This study |
| pWPI_HAHA-L-DGAT2_Puro | This study |
| pWPI_HAHA-L-DGAT2-del30-67_Puro | This study |
| pWPI_HAHA-L-DGAT2-del66-115_Puro | This study |
| pWPI_HAHA-L-DGAT2-mito4A_Puro | This study |
| pWPI_HAHA-L-DGAT2-del327-350_Puro | This study |
| pWPI_HAHA-L-DGAT2-insert-HA_Puro | This study |
| pLenti_CMV_TetR_BLR | [8] |
| pLenti CMV TO Puro DEST APEX2-V5 | [9] |
| pLenti_CMV-TO_HAHA-DGAT2_Puro | This study |
| pWPI_mRuby3_PKCe_C1a_C1b_Puro | This study |
| pWPI_mRuby3_PKCe-C1a-C1b-W264G_stop_Puro | This study |
| pCMV-Delta R8-74 | [10] |
| pczVSV-Gwt | [11] |

1. Reiss S, Rebhan I, Backes P, Romero-Brey I, Erfle H, Matula P, et al. Recruitment and activation of a lipid kinase by hepatitis C virus NS5A is essential for integrity of the membranous replication compartment. Cell Host Microbe. 2011 Jan 20;9(1):32–45.

2. Pietschmann T, Kaul A, Koutsoudakis G, Shavinskaya A, Kallis S, Steinmann E, et al. Construction and characterization of infectious intragenotypic and intergenotypic hepatitis C virus chimeras. Proceedings of the National Academy of Sciences. 2006 May 9;103(19):7408–13.

3. Ward JC, Bowyer S, Chen S, Fernandes Campos GR, Ramirez S, Bukh J, et al. Insights into the unique characteristics of hepatitis C virus genotype 3 revealed by development of a robust sub-genomic DBN3a replicon. J Gen Virol. 2020 Nov;101(11):1182–90.

4. Krieger N, Lohmann V, Bartenschlager R. Enhancement of Hepatitis C Virus RNA Replication by Cell Culture-Adaptive Mutations. J Virol. 2001 May;75(10):4614–24.

5. Backes P, Quinkert D, Reiss S, Binder M, Zayas M, Rescher U, et al. Role of Annexin A2 in the Production of Infectious Hepatitis C Virus Particles. J Virol. 2010 Jun;84(11):5775–89.

6. Lohmann V, Hoffmann S, Herian U, Penin F, Bartenschlager R. Viral and Cellular Determinants of Hepatitis C Virus RNA Replication in Cell Culture. J Virol. 2003 Mar;77(5):3007–19.

7. Laporte J, Malet I, Andrieu T, Thibault V, Toulme JJ, Wychowski C, et al. Comparative Analysis of Translation Efficiencies of Hepatitis C Virus 5′ Untranslated Regions among Intraindividual Quasispecies Present in Chronic Infection: Opposite Behaviors Depending on Cell Type. J Virol. 2000 Nov;74(22):10827–33.

8. Campeau E, Ruhl VE, Rodier F, Smith CL, Rahmberg BL, Fuss JO, et al. A versatile viral system for expression and depletion of proteins in mammalian cells. PLoS One. 2009 Aug 6;4(8):e6529.

9. Bersuker K, Peterson CWH, To M, Sahl SJ, Savikhin V, Grossman EA, et al. A Proximity Labeling Strategy Provides Insights into the Composition and Dynamics of Lipid Droplet Proteomes. Developmental Cell. 2018 Jan 8;44(1):97-112.e7.

10. Dull T, Zufferey R, Kelly M, Mandel RJ, Nguyen M, Trono D, et al. A Third-Generation Lentivirus Vector with a Conditional Packaging System. J Virol. 1998 Nov;72(11):8463–71.

11. Pietschmann T, Heinkelein M, Heldmann M, Zentgraf H, Rethwilm A, Lindemann D. Foamy virus capsids require the cognate envelope protein for particle export. J Virol. 1999 Apr;73(4):2613–21.
