# Supplementary material for: The triglyceride-synthesizing enzyme diacylglycerol acyltransferase 2 modulates the formation of the hepatitis C virus replication organelle
Source: PLoS Pathog. 2024 Sep 6;20(9):e1012509. doi: 10.1371/journal.ppat.1012509 (PMC11410266; doi:10.1371/journal.ppat.1012509)
Supplement: S2 Table — (DOCX) [file ppat.1012509.s010.docx]

S2 Table: Antibodies used in this study

| **Name or antigen targeted** | **Manufacturer** | **Order number** | **Species** | **Concentration (µg/mL) or dilution** | |
| --- | --- | --- | --- | --- | --- |
|  |  |  |  | **Immuno-fluorescence** | **Western blot** |
| **Primary antibodies** | | | | | |
| Calnexin | Abcam | ab22595 | rabbit | 1 | 0.5 |
| Calreticulin | Abcam | ab2907 | rabbit |  | 1 |
| CoxIV (F8) | Santa Cruz | sc-376731 | mouse | 0.4 |  |
| GAPDH | Santa Cruz | sc-47724 | mouse |  | 0.2 |
| HA tag | Novus Biologicals | NB600-362 | goat | 40 |  |
| HA tag (clone 16B12) | BioLegend | 901502 | mouse |  | 1 |
| HCV NS5A (9E10) | Gift from Charles Rice, produced by Cell Essentials | | mouse | 6.66 |  |
| Perilipin 2 (ADRP (B-6)) | Progen | 610102 | mouse | 1 |  |
| **Secondary antibodies** | | | | | |
| Goat anti-Mouse IgG Secondary Antibody, Alexa Fluor Plus 488 | Invitrogen | A32723 | goat, anti-mouse | 2 |  |
| Goat anti-Rabbit IgG Secondary Antibody, Alexa Fluor Plus 488 | Invitrogen | A32731 | goat, anti-rabbit | 2 |  |
| Goat anti-Mouse IgM Secondary Antibody, Alexa Fluor 568 | Invitrogen | A21043 | goat, anti-mouse | 2 |  |
| Chicken anti-Goat IgG Secondary Antibody, Alexa Fluor 647 | Invitrogen | A21469 | chicken, anti-goat | 2 |  |
| Goat anti-Mouse IgG Peroxidase Conjugate | Sigma Aldrich | A 4416 | goat, anti-mouse |  | 1/15,000 |
| Goat anti-Rabbit IgG Secondary Antibody, HRP | Invitrogen | G-21234 | goat, anti-rabbit |  | 1/15,000 |
