# Supplementary material for: The triglyceride-synthesizing enzyme diacylglycerol acyltransferase 2 modulates the formation of the hepatitis C virus replication organelle
Source: PLoS Pathog. 2024 Sep 6;20(9):e1012509. doi: 10.1371/journal.ppat.1012509 (PMC11410266; doi:10.1371/journal.ppat.1012509)
Supplement: S3 Table — (DOCX) [file ppat.1012509.s011.docx]

S3 Table: Reagents and chemicals used in this study

| **Name** | **Manufacturer** | **Product number or reference** |
| --- | --- | --- |
| 2-Mercaptoethanol | Carl Roth | 4227.3 |
| Blasticidine | Capricorn | BLA-5x |
| BSA Fraction V IgG Free Fatty Acid Poor | Fisher Scientific | 30036578 |
| BODIPY 493/503 | Fisher Scientific | D3922 |
| Carboxylmethylcellulose sodium | Sigma Aldrich | 21902 |
| Coelenterazin | PJK | 102171 |
| cOmplete, Mini Protease Inhibitor Cocktail | Roche | 4693124001 |
| Crystal violet | Carl Roth | T123.1 |
| Cycloheximide | Sigma Aldrich | 01810 |
| D(+)-Saccharose | Carl Roth | 4621.1 |
| Daclatasvir (BMS-790052) | Absource Diagnostic GmbH | S1482-0005 |
| DAPI | Life Technologies | D1306 |
| Dimethylsulfoxide | Carl Roth | A994.2 |
| D-Luciferine | PJK | 102112 |
| DMEM High Glucose (4.5 g/l) | Capricorn | DMEM-HA |
| Doxycycline (hyclate) | Biomol GmbH | Cay14422-1 |
| Fetal Bovine Serum (FBS) Advanced | Capricorn | FBS-11A |
| Fluoromount-G | Fisher Scientific | 15586276 |
| Formaldehyde | Fisher Scientific | 10459113 |
| HEPES 1M solution, Gibco | Fisher Scientific | 11560496 |
| LD540 | Gift from C. Thiele | [95] |
| L-Glutamine (200 mM), Gibco | Fisher Scientific | 11500626 |
| Lipofectamine 2000 | Invitrogen | 11668019 |
| MEM Non-Essential Amino Acids | Capricorn | NEAA-B |
| MTT (Thiazolylblau-tetrazoliumbromid) | Sigma Aldrich | M5655 |
| Oleic acid | Sigma Aldrich | O1008-1G |
| Paraformaldehyde, granulated | Carl Roth | 0335.3 |
| Penicillin/Streptomycin (100x) | Capricorn | PS-B |
| PF-06424439 (DGAT2i) | Sigma Aldrich | PZ0233 |
| Polyethylenimine, branched | Sigma Aldrich | 408727 |
| Puromycin, Powder100 mg | Capricorn | PUR-1X |
| Triton X 100 | Carl Roth | 3051.2 |
| Trypsin-EDTA (0.5 %), no phenol red, Gibco | Fisher Scientific | 15400054 |
