# Supplementary material for: The triglyceride-synthesizing enzyme diacylglycerol acyltransferase 2 modulates the formation of the hepatitis C virus replication organelle
Source: PLoS Pathog. 2024 Sep 6;20(9):e1012509. doi: 10.1371/journal.ppat.1012509 (PMC11410266; doi:10.1371/journal.ppat.1012509)
Supplement: S4 Table — (DOCX) [file ppat.1012509.s012.docx]

S4 Table: RT-qPCR primers used in this study.

| Primer name | Sequence | Final conc. (nM) | Reference |
| --- | --- | --- | --- |
| F-GAPDH | 5'-GAA GGT GAA GGT CGG AGT C-3' | 120 | [1] |
| R-GAPDH | 5'-GAA GAT GGT GAT GGG ATT TC-3' | 120 | [1] |
| F-DGAT2 | 5'-GGC TCA TCG CTG TGC TCT-3' | 120 | This study |
| R-DGAT2 | 5'-GGG GGT GGT ATC CAA AGA TAT AG-3' | 120 | This study |

1. Haid S, Windisch MP, Bartenschlager R, Pietschmann T. Mouse-Specific Residues of Claudin-1 Limit Hepatitis C Virus Genotype 2a Infection in a Human Hepatocyte Cell Line. Journal of Virology. 2010 Jan 15;84(2):964–75.
