# Supplementary material for: The triglyceride-synthesizing enzyme diacylglycerol acyltransferase 2 modulates the formation of the hepatitis C virus replication organelle
Source: PLoS Pathog. 2024 Sep 6;20(9):e1012509. doi: 10.1371/journal.ppat.1012509 (PMC11410266; doi:10.1371/journal.ppat.1012509)
Supplement: S5 Table — (DOCX) [file ppat.1012509.s013.docx]

S5 Table: RT-qPCR probes used in this study.

| Probe name | Sequence | 5‘ and 3‘ modifications | Final conc. (nM) | Reference |
| --- | --- | --- | --- | --- |
| GAPDH | 5'-CAA GCT TCC CGT TCT CAG CCT-3' | YYE – BHQ-1 | 200 | [1] |
| DGAT2 | 5'-TGG TCA GCA GGT TGT GTG TCT TCA CC-3' | FAM – BHQ-1 | 200 | This study |

1. Haid S, Windisch MP, Bartenschlager R, Pietschmann T. Mouse-Specific Residues of Claudin-1 Limit Hepatitis C Virus Genotype 2a Infection in a Human Hepatocyte Cell Line. Journal of Virology. 2010 Jan 15;84(2):964–75.
